# Expression interplay of genes coding for calcium-binding proteins and transcription factors during the osmotic phase provides insights on salt stress response mechanisms in bread wheat

**DOI:** 10.1007/s11103-024-01523-z

**Published:** 2024-11-01

**Authors:** Diana Duarte-Delgado, Inci Vogt, Said Dadshani, Jens Léon, Agim Ballvora

**Affiliations:** 1 INRES-Plant Breeding, University of Bonn, Bonn, Germany; 2 Research Group of Genetics of Agronomic Traits, Faculty of Agricultural Sciences, National University of Colombia, Bogotá, Colombia; 3 Present Address: Bean Program, Crops for Nutrition and Health, Alliance Bioversity International & International Center for Tropical Agriculture (CIAT), Cali, Colombia

**Keywords:** Sal stress transcriptome, Osmotic phase, Ca^2+^ signaling, EF-hand domain, Expression profiles, Polymorphisms, Promoter, 3ʹ-UTR

## Abstract

Bread wheat is an important crop for the human diet, but the increasing soil salinization is reducing the yield. The Ca^2+^ signaling events at the early stages of the osmotic phase of salt stress are crucial for the acclimation response of the plants through the performance of calcium-sensing proteins, which activate or repress transcription factors (TFs) that affect the expression of downstream genes. Physiological, genetic mapping, and transcriptomics studies performed with the contrasting genotypes Syn86 (synthetic, salt-susceptible) and Zentos (elite cultivar, salt-tolerant) were integrated to gain a comprehensive understanding of the salt stress response. The MACE (Massive Analysis of cDNA 3ʹ-Ends) based transcriptome analysis until 4 h after stress exposure revealed among the salt-responsive genes, the over-representation of genes coding for calcium-binding proteins. The functional and structural diversity within this category was studied and linked with the expression levels during the osmotic phase in the contrasting genotypes. The non-EF-hand category from calcium-binding proteins was found to be enriched for the susceptibility response. On the other side, the tolerant genotype was characterized by a faster and higher up-regulation of genes coding for proteins with EF-hand domain, such as RBOHD orthologs, and TF members. This study suggests that the interplay of calcium-binding proteins, WRKY, and AP2/ERF TF families in signaling pathways at the start of the osmotic phase can affect the expression of downstream genes. The identification of SNPs in promoter sequences and 3ʹ -UTR regions provides insights into the molecular mechanisms controlling the differential expression of these genes through differential transcription factor binding affinity or altered mRNA stability.

**Supplementary Information:**

The online version contains supplementary material available at 10.1007/s11103-024-01523-z.

## Key message

The fine-tuned expression of calcium-binding genes and transcription factors during the osmotic phase underlies the susceptibility and tolerance to salt stress responses of contrasting bread wheat genotypes.

**Supplementary Information:**

The online version contains supplementary material available at 10.1007/s11103-024-01523-z.

## Introduction

The increasing soil salinization worldwide is a major constraint for agriculture as most crops are salt-sensitive (Keshtehgar et al. 2013; Pessarakli and Szabolcs 2019). Bread wheat (*Triticum aestivum* L.) is a key staple crop for global food security that suffers significant reductions in yield and quality because of soil salinity (Zheng et al. 2009; Curtis and Halford 2014). The studies of complex traits in this species are challenging because of its intricate genome structure. This allohexapolyploid (2*n* = 6*x* = 42, AABBDD) contains three subgenomes of 16 Gb with 85% of repetitive sequences (Loginova and Silkova 2018; IWGSC 2018). Efforts from the International Wheat Genome Sequencing Consortium (IWGSC) have allowed the release of fully annotated and highly contiguous chromosome-level assemblies of diverse bread wheat lines (Montenegro et al. 2017; Shi and Ling 2018; IWGSC 2018; Borrill et al. 2019; Walkowiak et al. 2020; Zhu et al. 2021). The use of these assemblies has accelerated the dissection of complex traits as genes and genetic markers can be located in a physical context (Borrill et al. 2019). To achieve future global wheat demand, sequencing technologies can assist in the identification of molecular mechanisms, genes, and beneficial alleles for application in breeding programs aiming to develop cultivars with increased stress resilience (Varshney et al. 2021).

High soil salinity affects the plant growth because it leads to physiological drought conditions, ion toxicity, and cell oxidative damage (Tuteja 2007; Gupta and Huang 2014). According to the time of stress exposure, the plant growth response to salinity comprises the early osmotic and the late ionic phases. The osmotic phase is attributed to the reduced water potential in the rhizosphere because of the accumulation of salts in the soil and it is independent of the sodium accumulation in tissues (Ismail et al. 2014; Parihar et al. 2015; Julkowska and Testerink 2015). The primary consequence is the reduction of shoot growth and the production of new leaves because of the stomatal closure and the increase in leaf temperature (Roy et al. 2014). The signaling events in the osmotic phase include transient changes of intracellular Ca^2+^ that are sensed and provide crucial information for the acclimation response of the plants (Julkowska and Testerink 2015). The elevation of [Ca^2+^]_cyt_ involves multiple channels, channel-like proteins, and pathways including calcium-binding proteins regulating long-distance signaling and transcriptional reprogramming during stress (Himanen and Sistonen 2019; Malabarba et al. 2021). These calcium-binding proteins have a diverse affinity for calcium ions. This affinity combined with their architecture and sub-cellular localization can contribute defining their biological roles in stress response pathways (Ranty et al. 2016). In addition, transcription factors (TFs) are regulating the expression of stress-responsive genes involved in calcium-related signaling pathways (Bürstenbinder et al. 2017; Wan et al. 2018). TFs can be activated or repressed in signal cascades triggered by Ca^2+^ and calcium sensors (Galon et al. 2010).

The functional and structural diversity within the calcium-binding gene ontology term is broad and complex. Calcium-binding proteins are classified as EF-hand domain-containing or as non-EF-hand when other functional domains interact with Ca^2+^. Each one of these groups includes a variety of genes with diverse architectures and specific roles during susceptibility or tolerance responses to abiotic stresses (La Verde et al. 2018; Medvedev 2018; Mohanta et al. 2019). A better understanding of signaling pathways leading to abiotic stress tolerance in crop species can be obtained through the study of this gene category at the expression and sequence level. Natural variation in *cis*-regulatory sequences from calcium-binding genes can underlie the response to abiotic stresses by regulating mechanisms of expression in which TFs are involved (Ackermann et al. 2013; Cai et al. 2020).

Several approaches have been undertaken to gain insights into the biological functions of genes and related polymorphisms influencing salt stress response mechanisms in wheat. Genetic mapping analyses of salt stress response related traits have been performed in bi-parental populations (Dadshani 2018; Asif et al. 2021) and association panels from bread wheat (Oyiga et al. 2018, 2019; Javid et al. 2022; Hu et al. 2021). Some salt-tolerant induced mutants have been characterized at the biochemical, physiological (Lethin et al. 2022; Karimzadeh et al. 2023) and transcriptomic levels (Xiong et al. 2017). Transcriptomic analyses during the osmotic stress response from 6 to 72 h have been carried out in roots (Nakayama et al. 2022), as well as the expression reaction in leaves and roots have been compared from 1 to 48 h (Li et al. 2023). The availability of highly contiguous and fully annotated bread wheat genome assemblies has facilitated these studies (IWGSC 2018; Walkowiak et al. 2020).

Previously, the massive analysis of cDNA 3ʹ-ends (MACE) in leaves during the osmotic phase from 8 min to 4 h after stress exposure (ASE) in leaves, revealed crucial differences in the expression profiles from the genes included in over-represented categories from contrasting bread wheat genotypes. Transcripts annotated to encode proteins with calcium-binding domain were up- and down-regulated in the tolerant and susceptible genotypes, respectively (Duarte-Delgado et al. 2020). Within this ontology, genes coding proteins with EF-hand domain have been characterized in bread wheat at the phylogenetic and expression levels (Kaur et al. 2022; Li et al. 2022; Liu et al. 2022). Nevertheless, these studies have focused on the salt stress response after one hour and have omitted to analyze the groups of genes coding for calcium-binding proteins without EF-hand domain.

The very early salt stress reaction can be a critical phase in the acclimation performance of the plants (Pandolfi et al. 2017). The main goal of this research was to infer immediate osmotic stress response mechanisms related to the expression of genes coding for calcium-binding proteins and their interplay with TFs in leaves from contrasting bread wheat genotypes. This study contributes to the understanding of the influence of calcium-related transcript expression and their polymorphisms in the triggering of tolerance or susceptibility responses. This knowledge can be useful in the breeding of genotypes with improved resilience to salt stress effects. Some of the results within this manuscript are part of a PhD dissertation (Duarte-Delgado 2020).

## Materials and methods

### Clusters of time-course expression profiles

The expression of TFs and transcripts annotated to encode proteins with calcium-binding domain was retrieved from the data collected by Duarte-Delgado et al. (2020) in two contrasting bread wheat genotypes. In this study, a comparative transcriptomic analysis was performed during the early osmotic phase at 8, 15, 30 min, and 4 h ASE in leaves. The time points for this experiment were selected based on the key turning points observed in the photosynthesis rate curve reported by Dadshani (2018) using a gas exchange system. Time-course expression profiles for salt-responsive transcripts were identified from over-represented gene ontologies in the differential genotypes. This transcriptomics data is deposited in the BioProject from the Sequence Read Archive of the National Center for Biotechnology Information with accession number PRJNA549411. The genotypes studied consisted of the elite German winter wheat cultivar Zentos (salt-tolerant) and the synthetic genotype Syn86 (salt-susceptible), which are also contrasting parents used for advanced backcross QTL studies (Kunert et al. 2007; Dadshani 2018).

Clusters of time-course expression profiles were identified for the genes annotated to encode proteins with calcium-binding domain through the visualization of the patterns of up- or down-regulation at 8, 15, 30 min, and 4 h ASE in the two genotypes. The expression profiles from the TFs were clustered according to the family type and compared for the two genotypes. The LOESS (locally estimated scatterplot smoothing) option from the *ggplot2* library from R software (R Core Team 2021) was used to fit a curve representing the expression tendency of the members of each calcium-binding cluster and each TF family, as indicated by Duarte-Delgado et al. (2020).

### Classification of calcium-binding proteins coded by salt-responsive genes

The characterization of the functional calcium-binding domains from the proteins coded by salt-responsive genes was performed using the Interpro classification of protein sequences (Mitchell et al. 2019) available in the RefSeqv1.0 annotation of the Chinese Spring wheat genome (Alaux et al. 2018). The proteins encoded by these genes were classified as EF-hand or non-EF-hand according to the calcium-binding domain type present.

### Phylogenetic analysis of EF-hand proteins and transcription factors

To deduce the functional properties and the putative involvement of salt-responsive genes coding for proteins with EF-hand domain and TFs in regulatory networks related to stress response, a comparative phylogenetic analysis was performed with amino acid sequences from *Arabidopsis thaliana.* This step is crucial to compare the sequences from the different organisms, which can provide insights into functional relationships and evolutionary history. This analysis enabled the classification of the wheat EF-hand proteins without additional functional domains as calmodulin (CaM), calmodulin-like (CML), or calcineurin B-like (CBL) type. The EF-hand proteins with kinase domain were distinguished as members of the CPK (calcium-dependent protein kinases) family through the comparison with peptides sequences from CBL-interacting protein kinase (CIPK), CPK-related kinase (CRK), calcium/calmodulin-dependent protein kinase (CCaMK) and CPK members from *A. thaliana* (Ho 2015; Shi et al. 2018; Mohanta et al. 2019). Three NADPH oxidases (respiratory burst oxidase homologs, RBOHs) were also analyzed among the salt-responsive genes in wheat. These proteins with two EF-hand motifs produce localized reactive oxygen species (ROS) bursts from the outer of the plasma membrane. RBOHs are involved in the signaling to induce responses as stomatal closure upon stress sensing (Chapman et al. 2019; Shen et al. 2020). The genes from WRKY and APETALA2/Ethylene-Responsive-Element-Binding protein (AP2/ERF) families were further characterized and classified into subfamilies, as they were the most abundant TF families in the transcriptomic analysis.

A multiple sequence alignment was performed with MAFFT (Katoh et al. 2019) to identify conserved regions and patterns among the sequences from the different organisms. Then, a phylogenetic tree analysis was conducted with MEGA X (Kumar et al. 2018) through the Neighbour-Joining method which is a distance-based approach that estimates relationships among sequences based on the number of differences observed in the alignments and the branch length at each stage of clustering (Saitou and Nei 1987). To enhance the reliability of the defined phylogenetic relationships, a bootstrap analysis with 3000 replicates was conducted (Hedges 1992) to infer consensus trees for salt-responsive CaM/CML, CPK, RBOH, AP2/ERF, WRKY and the corresponding reference proteins from *A. thaliana*. This analysis involved randomly resampling the original dataset multiple times and constructing separate trees for each resample to calculate bootstrap values. Bootstrap values greater than 50 were considered to represent highly reliable clusters (Lemoine et al. 2018).

### RT-qPCR analysis of calcium-binding genes

The real-time quantitative PCR (RT-qPCR) analysis validated the expression levels of the genes in leaves and expanded the analysis to the roots for two members of the RBOH family, *TraesCS5B02G299000* and *TraesCS4D02G324800*. The RT-qPCR data from stressed leaves for the gene *TraesCS5D02G238700* coding for a CML protein (Duarte-Delgado et al. 2020), was complemented with expression measurements in roots using the reported primers. Seeds of Zentos were purchased from Syngenta Seeds GmbH (Bad Salzuflen, Germany), while Syn86 seeds have been multiplied after their supply by Lange and Jochemsen (1992). Seedlings were in a growth chamber (20 ± 2 °C, 50 ± 5% humidity,12 h photoperiod) in the hydroponic system proposed and detailed by Dadshani (2018). Eight days after seed germination in Petri boxes with filter paper and distilled water, healthy seedlings were transferred to sponges located in panels over hydroponic boxes with 170 L of nutrient solution.

A salt treatment of 150 mM NaCl was applied to two-weeks-old seedlings that were sampled at 8 min, 15 min, 30 min, and 4 h ASE, according to the photosynthesis turning time points previously identified (Dadshani 2018; Duarte Delgado et al. 2020). The control conditions corresponded to untreated hydroponics boxes containing plants that were harvested simultaneously with the stressed plants at the same time points. A biological replicate consisted of the whole root or all the leaves from a single plant. Three biological replicates were sampled from each organ, genotype, treatment, and time point. Leaves and roots were separated with scissors and immediately frozen in liquid nitrogen in 15 ml falcon tubes, followed by tissue homogenization with liquid nitrogen, mortar, and pestle. Total RNA isolation from 200 mg of grounded tissue was performed with the RNeasy plant mini kit (Qiagen, Hilden, Germany) and the cDNA synthesis with the First Strand cDNA Synthesis Kit (Thermo Scientific, Waltham, MA, USA) as described by Duarte-Delgado et al. (2020).

To design subgenome-specific primers for the salt-responsive RBOH genes (Table 1) the web-based tool GSP was used (Wang et al. 2016). Denaturation at 95 °C/7 min followed by 40 cycles at 95 °C/10 s, annealing temperature (Ta) for 30 s, 72 °C/30 s, and fluorescence acquisition temperature (Tfa) for 30 s were the cycling conditions used for RT-qPCR in a SDS-7500 Sequence Detection System device (Applied Biosystems, Waltham, MA, USA). Ta and Tfa conditions specific for each primer pair are listed in Table 1. The RT-qPCR reaction of 10 μl consisted of 0.25 μM of each primer, 5 µl of Luna® Universal qPCR Master Mix (Biolabs, Ipswich, MA, USA) and 2 μl from 1:20 diluted cDNA template.

**Table 1:** RT-qPCR primers of target genes with their respective annealing (Ta) and fluorescence acquisition (Tfa) temperatures

Gene	Sequence 5ʹ–3ʹ	Ta (°C)	Tfa (°C)	Amplicon	Efficiency (slope)	
				size (bp)	*Ef1.1*	*Ef1.2*
*TraesCS4D02G324800*	F-CTTATACTTGTGAGTAGAGCAAC	60	76	239	**0.14**	0.16
	R-TTTCTTAATCAACATCCATGGTC					
*TraesCS5B02G299000*	F-GCACCAGCGTCTACGAGGAA	59	84	221	nd^a^	**0.04**
	R-CACCGGTGCTGATAAAGGAG					

The amplification efficiency assessment was performed with the reference genes *Ef1.1* and *Ef1.2* (Oyiga et al. 2018, 2019) and was compared. Bold values represent the internal control selected for each target gene primer, according to the efficiency

^a^The reference gene was not detected under the cycling and fluorescence acquisition conditions established for the target gene

The amplification efficiencies of the internal control primers *TaEf-1.1* and *TaEf-1.2* (Oyiga et al. 2018, 2019) and each target gene were compared (Duarte-Delgado et al. 2020). For melting curve analysis (Supplementary Fig. S1), a cycle of 95 °C for 10 s, Ta for 30 s, and 95 °C for 15 s was applied to PCR products. Because of the greater amplification efficiency (Table 1), *TaEf-1.1* was the reference gene selected for the analysis of *TraesCS4D02G324800*. This reference gene was not detected in the RT-qPCR of *TraesCS5B02G299000*, because the Tfa defined in the cycling conditions (Table 1) was higher than the melting temperature from *TaEf-1.1* (82.5 °C; Duarte-Delgado et al. 2020). The other reference gene, *TaEf-1.2*, was therefore selected for the expression analysis in this case. The melting curves originated from the amplification of *TraesCS5B02G299000* revealed unspecific peaks (Supplementary Fig S1b). To avoid the contribution of the fluorescence from the unspecific products to the quantification, the temperatures of Tfa (Table 1) were adjusted to a temperature below the melting temperature (Tm) of the target product, as suggested by Klein et al. (2004).

The ∆∆Ct method (Livak and Schmittgen 2001) was used to quantify the relative expression of the selected genes with the average Ct values of three technical replicates. A one-sample single-tailed t-test (*p* < 0.05) was implemented in the 2^−∆∆*Ct*^ values, as detailed by Duarte-Delgado et al. (2020), to define whether the transcripts were up- or down-regulated upon stress in each time point, genotype, and organ. To determine if the mean relative expression values from both genotypes and organs were significantly different at each time point, a two-sample two-tailed t-test (*p* < 0.05) was used to compare the 2^−∆∆*Ct*^ values from the same organ in the two genotypes and the two organs within the same genotype.

### Promoter sequence analyses

The promoter from *TraesCS2D02G173600, TraesCS5D02G238700,* and *TraesCS5B*02G299000 genes were analyzed by Sanger-based sequencing after PCR amplification. Subgenome-specific primers were designed (Table 2) as described in the previous section based on the sequence of *ca.* 2000 bp upstream the start codon of the genes identified using the genome browser tool from the IWGSC RefSeq v1.0 (Alaux et al. 2018). The amplification conditions were adjusted using DNA isolated from leaves with the Plant DNA mini kit (VWR, Darmstadt, Germany). Thus, 100 ng of DNA template in 25 µl of 1 × One Taq Standard Buffer (Biolabs, Ipswich, MA, USA), 0.2 mM dNTPs, and 0.2 µM of each primer were amplified with 0.5 units of One Taq DNA polymerase (Biolabs, Ipswich, MA, USA). Cycling conditions were established with an initial denaturation step at 95 °C/2 min followed by 40 cycles at 95 °C/45 s, Ta for 45 s (specified in Table 2), extension at 72 °C/1 min per kbp, and a final extension step at 72 °C/5 min. The amplicons were visualized on 1.5% (w/v) agarose gels stained with peqGreen (0.04 µl/ml; VWR, Darmstadt, Germany). PCR products were purified with Purelink Quick PCR kit (Invitrogen, Waltham, MA, USA) and sequenced at Eurofins Genomics (Ebersberg, Germany) with an ABI 3730xl DNA Analyzer System.

**Table 2:** Primers designed to amplify the promotor regions of target genes studied by RT-qPCR, with their respective annealing temperatures (Ta)

Gene	Sequence 5ʹ–3ʹ	Ta (°C)	Amplicon size (bp)
*TraesCS2D02G173600*	F-AATAGGGTATTAGGAATGTCAC	58	1485
	R-GAATCTCGTCCCTTTTTGTTC		
*TraesCS5B02G299000*	F-TAGATGGCTGGGAAATGAAAGC	60	1048
	R-TCTTG GTACCGCACGAGAAA		
	F-AGAAATCTCATTACCAGGCAAAA	60	975
	R-CTACTACGTGAGCCTAATCCT		
*TraesCS5D02G238700*	F-GGTGCTTGCTTCTTGATCGT	60	1454
	R-GCTAATTATGTGTGCGAGCG		

Polymorphisms in the contrasting genotypes were identified using the pairwise alignment tool from Bioedit version 7.2.5 (Hall et al. 1999). The use of the PlantTFDB (v 5.0) database allowed the prediction of TF families with recognition motifs in the promoter sequences (Jin et al. 2017). TF families binding to motifs containing polymorphic sites were recognized as putative regulators of gene expression when *q-*value < 0.05.

### MACE-based SNP identification and miRNA binding site analysis

SNPs in salt-responsive genes coding for calcium-binding proteins were identified through the examination of the MACE reads collected for the transcriptomics analysis (Duarte-Delgado et al. 2020). These polymorphisms contributed to predict variations in miRNA binding sites that can be linked to the differences in expression observed in the contrasting genotypes (Võsa et al. 2015). The unique mapped reads from the MACE libraries of each genotype were merged for SNP identification using SAMtools (Li et al. 2009). Variant calling was performed with *samtools mpileup* followed by *bcftools call* commands from SAMtools, as described by Schneider et al. (2022). To retain high-confidence SNP calls, we established the following conditions: the minimum Phred base quality in the variant site was 25, the minimum mapping quality from the read was 20 and variants were represented in at least 10 reads from the same genotype. The psRNAtarget tool allowed the prediction of miRNA binding sites in the regions adjacent to the polymorphisms, based on known regulatory interactions in model species (Dai et al. 2018). Sequences of 10 nucleotides upstream and downstream the polymorphisms were retrieved for this analysis.

### Protein–protein interaction prediction

Previous studies have reported the phosphorylation of RBOH proteins by kinases with CPK annotation in Arabidopsis to regulate TFs under abiotic stresses (reviewed in Yip-Delormel and Boudsocq 2019). The possible interactions among three RBOH and seven CPK proteins coded by salt-responsive genes identified in this study were predicted with the STRING server using the bread wheat proteins database included (Szklarczyk et al. 2023). In the case of various isoforms possible, the longest coded protein was selected. Predictions scores greater than 0.7 are considered high and represent plausible protein–protein interactions that can be prioritized for validation analyses in the wet laboratory. The interaction network was visualized with the *ggraph* package (Pedersen 2024) implemented in R software (R Core Team 2021).

## Results

### Diversity of calcium-binding proteins encoded by salt-responsive genes during the osmotic phase

**Fig. 1:**
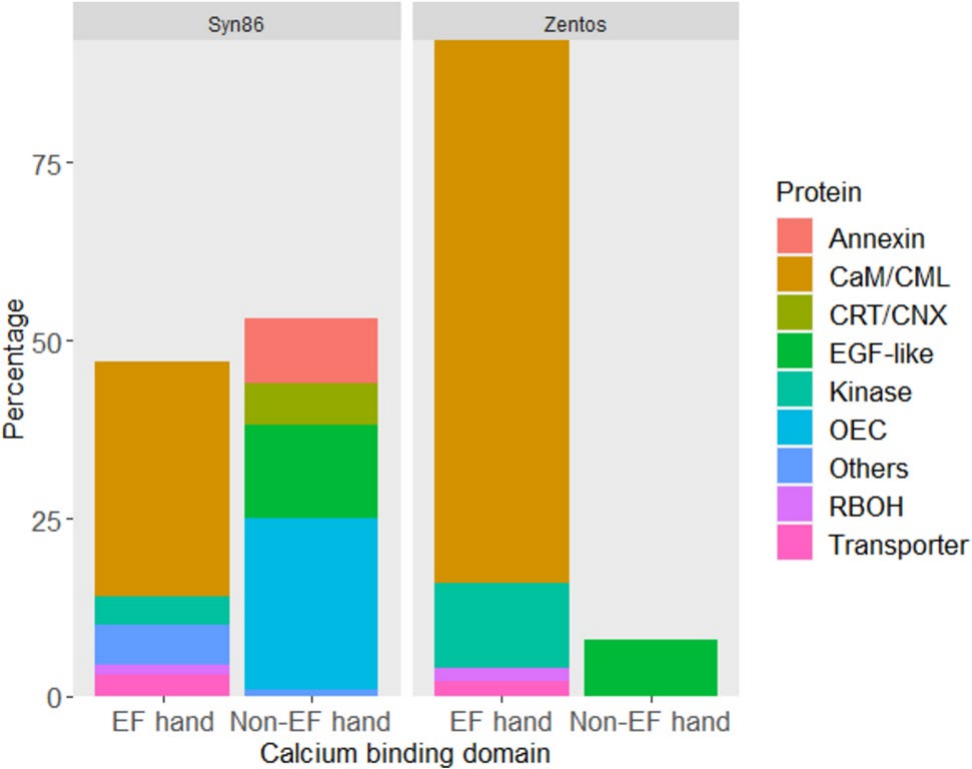
Distribution of categories of calcium-binding proteins with EF-hand and non-EF-hand domain coded by the salt-responsive genes identified in the contrasting genotypes. CaM/CML (calmodulin/calmodulin-like), CRT/CNX (calnexin/calreticulin), EGF-like (epidermal growth factor-like), OEC (oxygen-evolving complex) and RBOH (respiratory burst oxidase homolog)

The MACE analysis of leaves from 8 min to 4 h after salt stress exposure (ASE), detected salt-responsive genes annotated to encode proteins with different sets of calcium-binding domains in the contrasting genotypes. The proportion of salt-responsive genes coding EF-hand and non-EF-hand domains was determined in the contrasting genotypes (Fig. 1). The tolerant genotype showed 92% of genes coding for calcium-binding proteins containing EF-hand domain. From this, 76% did not have any other functional domain and 12% contained a kinase domain. The non-EF-hand group was a minority (8%) with genes having an Epidermal Growth Factor (EGF)-like domain (Fig. 1; Supplementary Table S1). A higher percentage of genes coding non-EF-hand domains was observed in Syn86 (53%). This genotype also showed increased functional and structural diversity within the EF-hand and non-EF-hand groups (Fig. 1; Supplementary Table S2). Most of the non-EF-hand type proteins belonged to the oxygen-evolving complex (OEC; 24%) followed by proteins with EGF-like domain (13%). The EF-hand group consisted mainly of CaM/CML proteins that lacked any other functional domain (33%) followed by EF-hand proteins with other additional domains (5.5%). In the latter group, proteins such as caleosins (1.5%) and phosphoglycolate phosphatases (2%) were included (Supplementary Table S2).

**Fig. 2:**
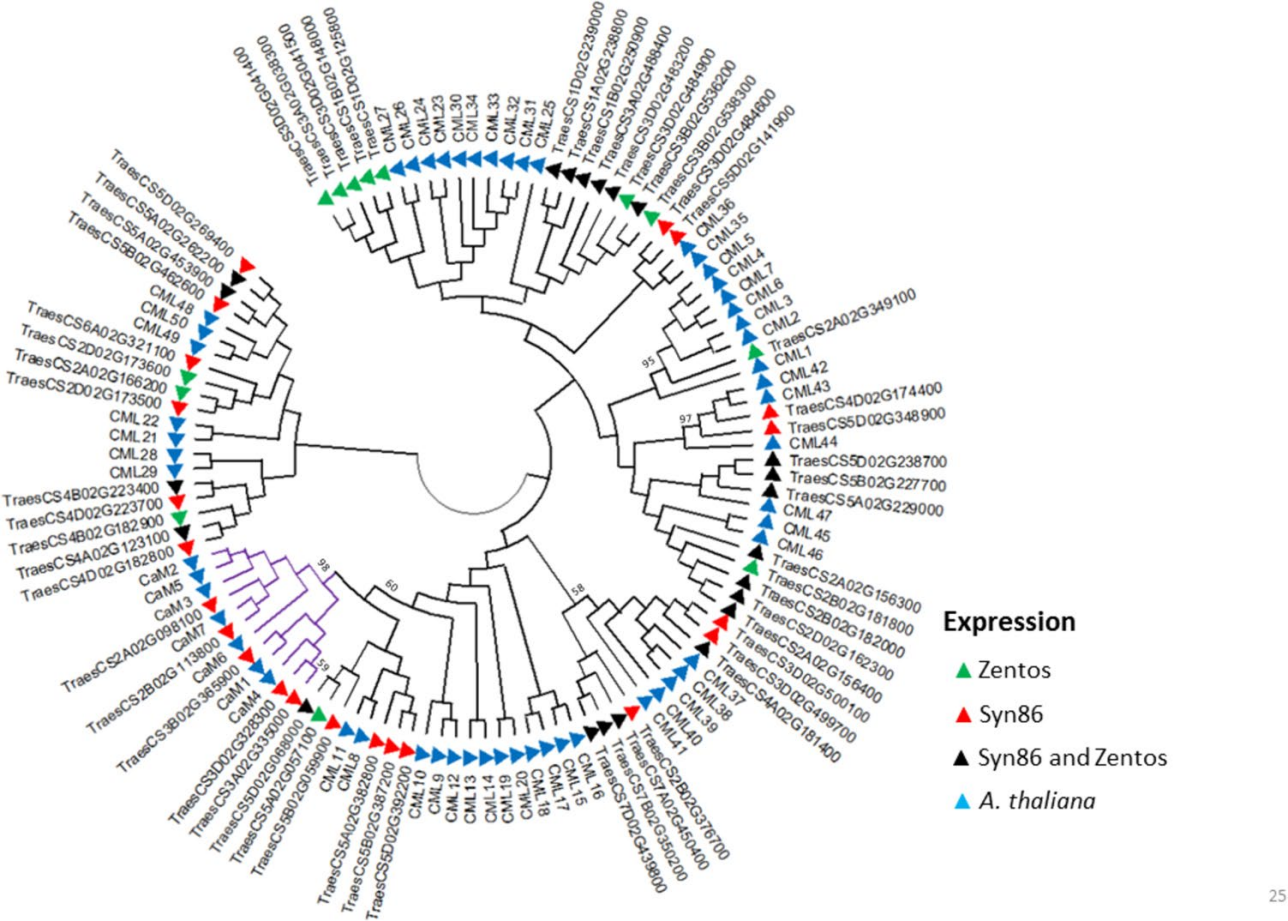
Dendrogram of CaMs (purple) and CMLs (black) amino acid sequences from *Arabidopsis thaliana* and coded by the corresponding salt-responsive genes from Zentos and Syn86 indicated by the colored triangles. The consensus phylogenetic tree was constructed with MEGA X (Kumar et al. 2018) using the Neighbour-Joining method and through a bootstrap analysis of 3000 replicates. Clusters of wheat and *Arabidopsis* proteins with bootstrap values > 50 are shown

The phylogenetic analysis of EF-hand domain proteins revealed the similarities and the clustering with CaM/CML, CBL, CPK, and RBOH members from *A. thaliana.* The subgroup of CaM had the highest statistical support (98%) in the CaM/CML analysis and included five salt-responsive genes from Syn86 (Fig. 2). From all proteins containing an EF-hand domain, 57 (72%) corresponded to the CML type. Mostly low bootstrap values (< 50%) were observed in the corresponding clusters (Fig. 2). This analysis therefore revealed orthologous relationships with low statistical support among wheat and *Arabidopsis* CML sequences. The salt-responsive *TraesCS1B02G370900* from Syn86 was the only transcript identified from the CBL type and is orthologous to *CBL8*.

**Fig. 3:**
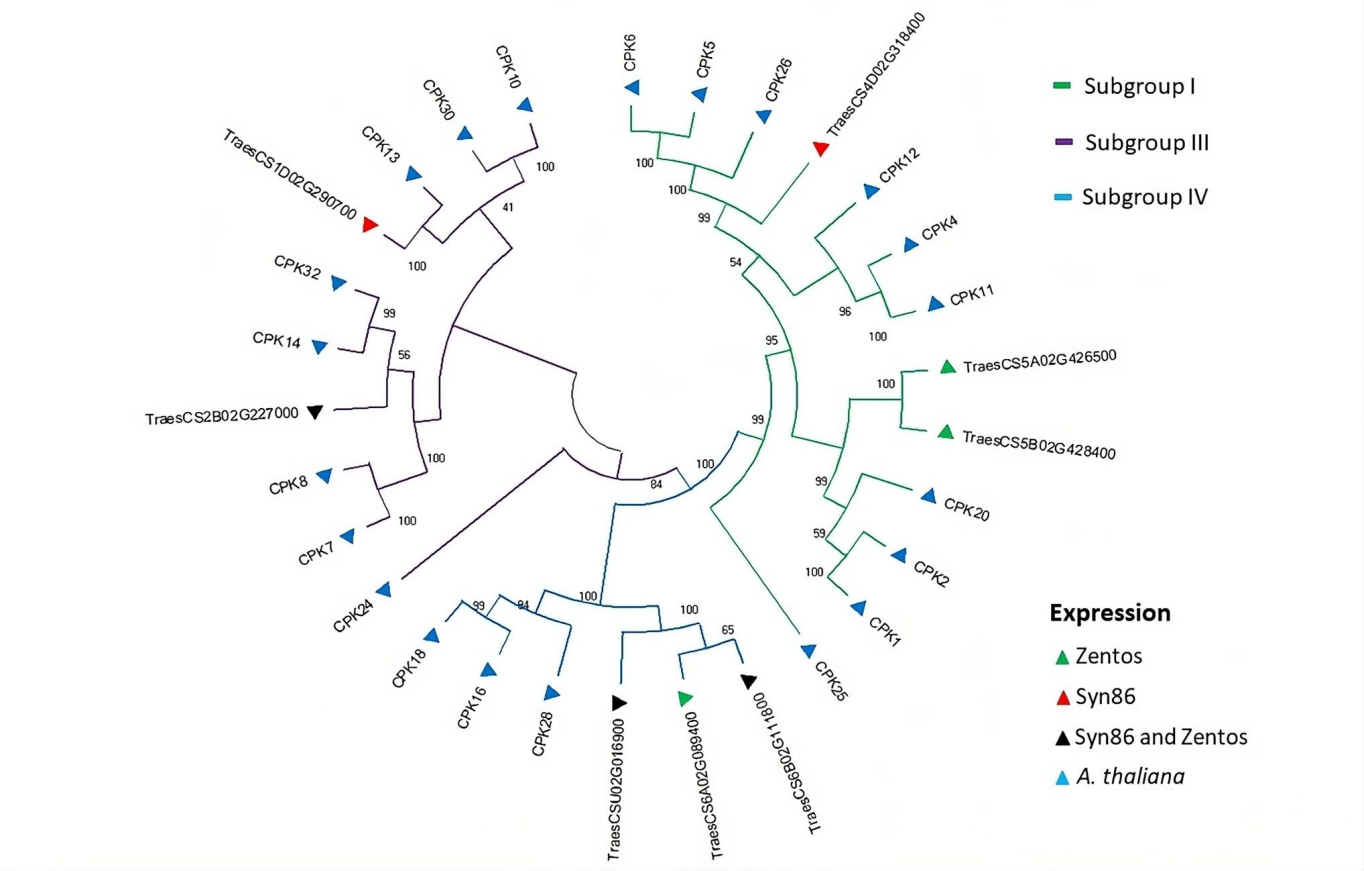
Dendrogram of CPKs amino acid sequences from *Arabidopsis thaliana* and coded by the corresponding salt-responsive genes from Zentos and Syn86 indicated by the colored triangles. The consensus phylogenetic tree was constructed with MEGA X (Kumar et al. 2018) using the Neighbour-Joining method and through a bootstrap analysis of 3000 replicates. The subgroups indicated by colors in the branches were defined according to the classification suggested by Yip-Delormel and Boudsocq (2019)

**Fig. 4:**
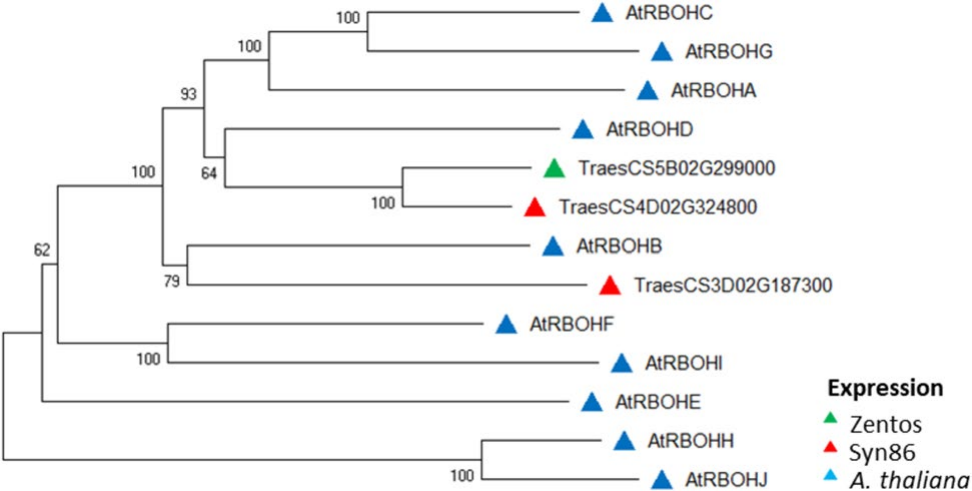
Dendrogram of RBOHs amino acid sequences from *Arabidopsis thaliana* and the corresponding salt-responsive genes from Zentos and Syn86 indicated by the colored triangles. The consensus phylogenetic tree was constructed with MEGA X (Kumar et al. 2018) using the Neighbour-Joining method and through a bootstrap analysis of 3000 replicates

All the differentially expressed genes coding EF-hand proteins with kinase domain were from the CPK type, and clustered with good statistical support in subgroups I, III, and IV based on the classification proposed by Yip-Delormel and Boudsocq (2019) (Fig. 3). Three wheat genes were found in the subgroup I. From them, two homoeologous genes located in chromosomes 5A and 5B were up-regulated in Zentos at 15 min and were included in a cluster with *CPK1*, *CPK2,* and *CPK20*. The subgroups III and IV contained two and three wheat genes, respectively (Fig. 3). Similarly, the analysis of RBOH proteins revealed clusters with good statistical support (Fig. 4). From three proteins of wheat, two clustered with RBOHD and the third presented similarity with RBOHB. The wheat RBOHD orthologs were up-regulated, *TraesCS4D02G324800* in Zentos at 15 min and *TraesCS5B02G299000* in Syn86 at 30 min.

**Fig. 5:**
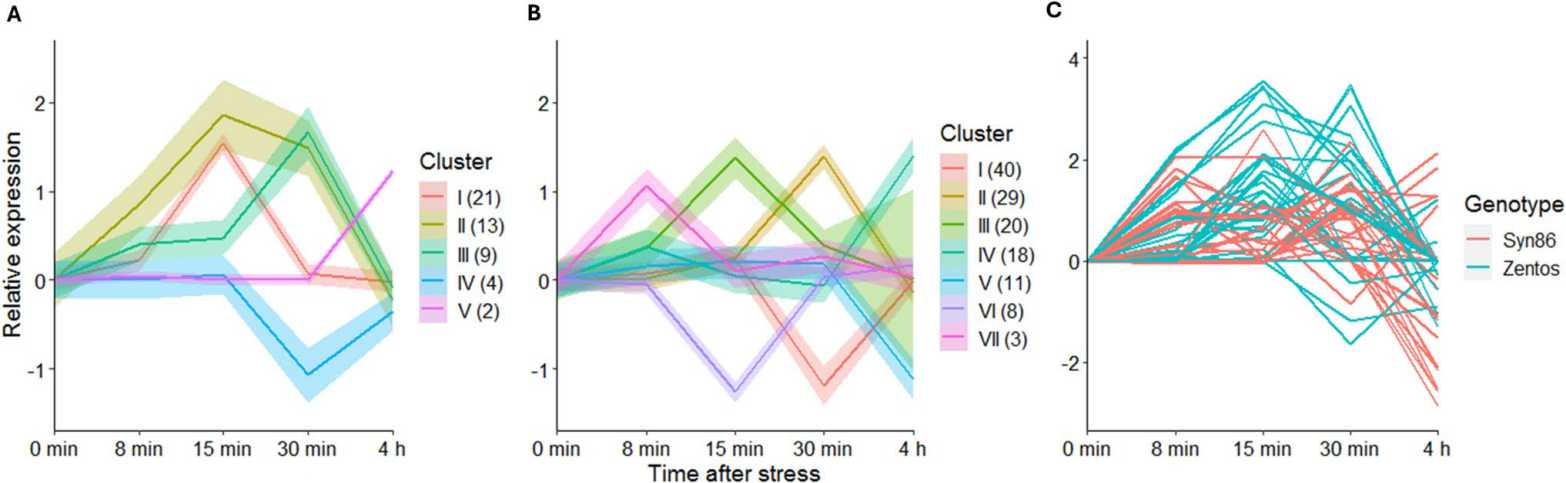
Expression profiles at the osmotic phase of salt-responsive genes annotated with calcium-binding domain, grouped in clusters represented by Roman numbers for Zentos (**A**) and Syn86 (**B**). The number of genes in each cluster is in parenthesis. A curve was fitted to represent the expression tendency of the transcripts from each group and the shadows indicate the standard error of the relative expression values. Expression profiles of 27 calcium-binding transcripts identified in both genotypes (**C**)

Clusters of time-course expression profiles were determined for the salt-responsive genes coding for calcium-binding proteins from each genotype (Fig. 5). The tolerant genotype showed five clusters, the main one with genes up-regulated at 15 min followed by a group of transcripts up-regulated both at 15 and 30 min (Fig. 5A). Seven clusters were defined in the susceptible genotype. Clusters I and II grouped the largest number of genes with down- and up-regulated transcripts at 30 min, respectively (Fig. 5B). Cluster I contained mainly OEC proteins and annexins, while the cluster II included EF-hand domain, EGF-like and calnexin/calreticulin (CNX/CRT) proteins. The two genotypes shared 27 transcripts annotated to encode proteins with calcium-binding domain, 22 of which corresponded to the CML type. The up-regulated transcripts across the early osmotic stress response showed greater relative expression values in Zentos compared to Syn86 (Fig. 5C). At 4 h ASE, 13 of these genes were down-regulated in the susceptible genotype. The time-course expression values, the annotations, and the clusters of the genes encoding calcium-binding proteins are included in Supplementary Tables S1 and S2.

### Time-course expression of EF-hand genes in roots and leaves by RT-qPCR

**Fig. 6:**
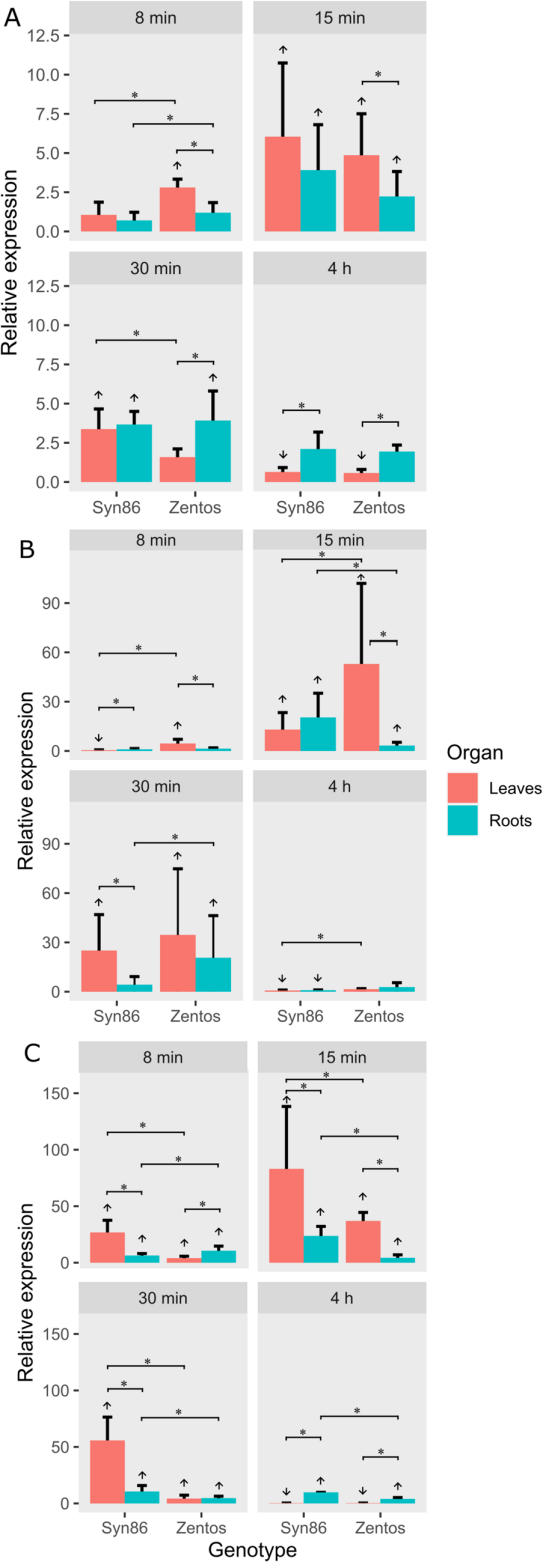
Relative expression values calculated with the ΔΔCt method (Livak and Schmittgen 2001) in leaves and roots from Syn86 and Zentos during the osmotic phase. **A** *TraesCS4D02G324800 (RBOH)*, **B** *TraesCS5B02G299000 (RBOH)* and **C** *TraesCS5D02G238700 (CML)* expression. Mean relative expression values > 2.0 or < 1.0 (*p* < 0.05) indicated up-regulation (**↑**) or down-regulation (**↓**) of genes, respectively. Asterisks represent significant differences in the mean values from the two genotypes in the same organ and differences in the mean values from the two organs in the same genotype using pairwise *t-*tests (*p* < 0.05). Expression of leaves in **C** was extracted from Duarte-Delgado et al. (2020)

The time-course expression profiles of one CML member (*TraesCS5D02G238700*) and two members from the RBOH family (*TraesCS5B02G299000* and *TraesCS4D02G324800*) were analyzed by RT-qPCR in leaves and roots. The time-course analysis of the RBOH family members indicated that transcripts were up-regulated earlier in leaves than in roots. The measurements of *TraesCS4D02G324800* in leaves revealed the transcript up-regulation in Zentos at 8 min and in both genotypes at 15 min ASE. At this later time point, the relative expression values were the highest and there were non-significant differences in the mean values from the two genotypes. At 30 min, the gene was only up-regulated in Syn86, while at 4 h it was down-regulated in both genotypes (Fig. 6A). The analysis in roots showed the transcript up-regulation in Syn86 at 15 min and in both genotypes at 30 min ASE without significant differences (Fig. 6A). The mean expression values from the two organs were different in the tolerant genotype across all the time points.

The relative expression values from the other member of the RBOH family, *TraesCS5B02G299000*, were higher (Fig. 6B). The analysis in leaves evidenced the opposite regulation of the expression of this transcript in the contrasting genotypes at 8 min ASE, with up-regulation in Zentos and down-regulation in Syn86. Later, at 15 and 30 min ASE the gene was up-regulated in both genotypes. At 15 min, the tolerant genotype registered a statistically significant higher expression than the susceptible (Fig. 6B). In roots, this transcript was up-regulated in both genotypes at 15 min and the relative expression value was significantly higher in the susceptible one. Later at 30 min, the gene was only up-regulated in Zentos (Fig. 6B). The expression analysis at 4 h unveiled the down-regulation of the gene in both organs from the susceptible genotype, while in the tolerant it was not stress-responsive.

The study of *TraesCS5D02G238700* in roots complemented the analysis performed in leaves (Duarte-Delgado et al. 2020) and indicated the up-regulation of the transcript across all time points with mean relative expression values differing significantly in both genotypes. The mean expression values calculated for the organs were significantly different in all time points, except for Zentos at 30 min. At 15 min ASE, Syn86 registered the highest mean relative expression value (Fig. 6C).

### Diversity of salt-responsive transcription factors during the osmotic phase

**Fig. 7:**
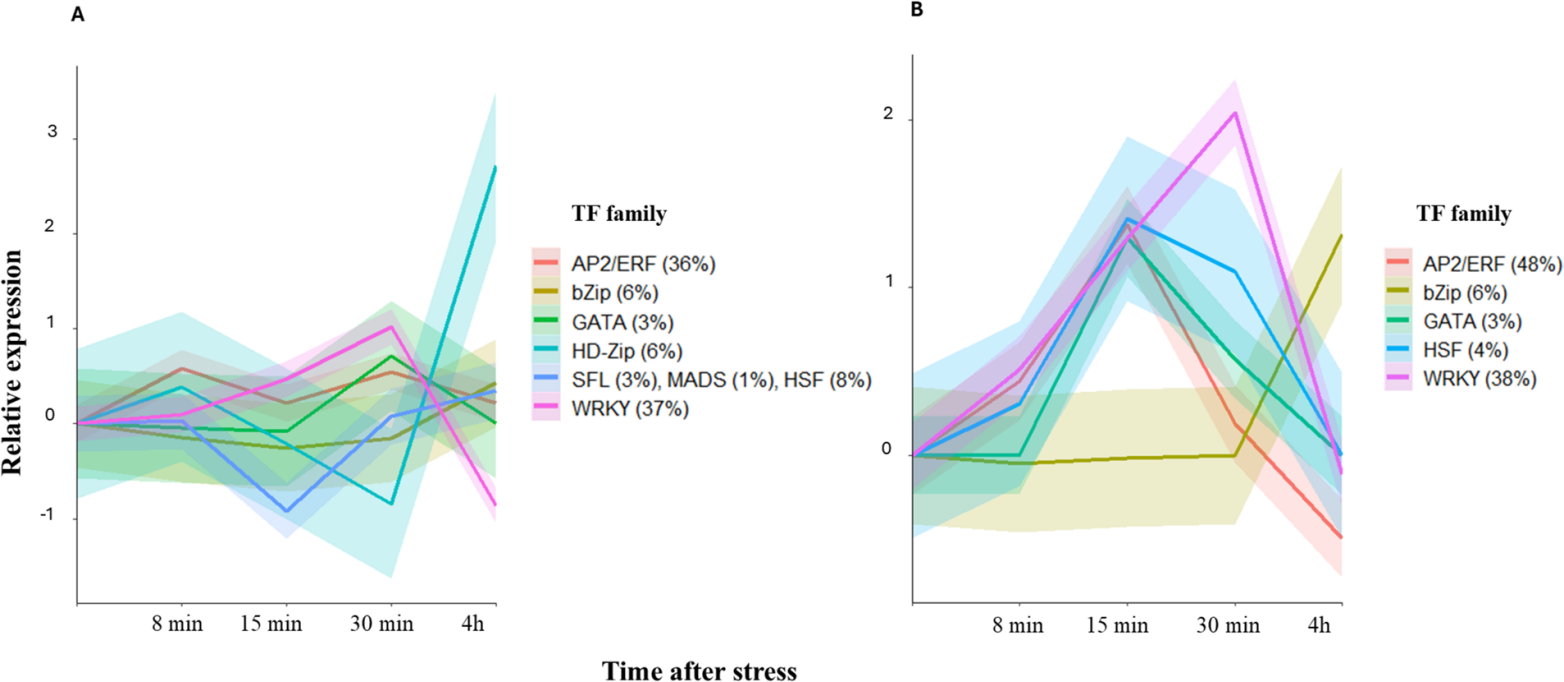
Expression patterns of salt-responsive transcription factor (TF) families in Syn86 (**A**) and Zentos (**B**) during the osmotic phase. The frequencies of the families are indicated in parentheses. A curve was fitted to represent the expression tendency of the members of each TF family and the shadows indicate the standard error of the relative expression values

To gain insights into the interplay of calcium-related signaling pathways and transcriptional regulation during stress response, the expression profiles from the salt-responsive TF families were characterized in Syn86 and Zentos. This analysis revealed the salt stress effect on the up-regulation of members from the AP2/ERF, WRKY, bZip, and GATA families in the two genotypes. The families HD-Zip, HSF, SFL, and MADS were down-regulated in Syn86 at 15 or 30 min ASE. Most TFs were up-regulated in Zentos at 15 min and presented greater relative expression values than those observed in Syn86 (Fig. 7). AP2/ERF and WRKY were the most expressed families in the two genotypes under stress conditions (Fig. 7). The expression profiles from the AP2/ERF family in Syn86 showed two peaks, with up-regulated genes at 8 and 30 min. On the other side, the members of this family in Zentos revealed higher expression values during one peak at 15 min (Fig. 7). The members of the WRKY family presented an up-regulation peak at 30 min in both genotypes, with the greatest expression values in the tolerant. At 4 h ASE the down-regulation of these genes is observed in Syn86 (Fig. 7). The members of the GATA family were up-regulated at 15 min in Zentos, and later at 30 min in Syn86. The fitted curves evidenced the up-regulation of members of the bZip family at 4 h ASE in both genotypes and Zentos unveiled far-reaching relative expression values. Finally, the HSF family revealed opposite expression values in the contrasting genotypes, with down- and up-regulation in Syn86 and Zentos, respectively (Fig. 7). The relative expression values of the salt-responsive TF family members during the osmotic phase are listed in Supplementary Table S3.

A comparative phylogenetic analysis of the salt-responsive WRKY and AP2/ERF genes facilitated their assignation to defined subfamilies to better understand their roles in the salt stress response. From 65 WRKY genes, 54% were expressed in both genotypes and 34% were exclusive from Syn86. Subfamily I contained 39% of the genes followed by subfamilies III (32%) and II (29%) (Supplementary Fig. S2). Within the later subfamily, the IIc harbored most of TFs (23%), while only three genes were identified in group IId (5%).

The analysis of 84 salt-responsive AP2/ERF transcripts assigned 57% of them to the ERF (Ethylene-Responsive-Element-Binding protein) subfamily and 38% to the DREB (Dehydration Responsive Element-Binding) subfamily (Supplementary Fig. S3). The ERF subfamily included 44% of transcripts with specific expression in Syn86 and 35% that were expressed in both genotypes. Among the salt-responsive DREB genes, 53% were expressed exclusively in Zentos followed by 28% of them expressed in both genotypes. *TraesCS2B02G542400*, *TraesCS5A02G473800*, *TraesCS1A02G058400* were assigned to the AP2 (APETALA2) subfamily according to the classification proposed by Zhao et al. (2019) for bread wheat. *TraesCS1B02G392300* was the only member from the RAV (Related to ABI3/VP) subfamily (Supplementary Fig. S3).

### Identification of transcription factor binding sites

**Fig. 8:**
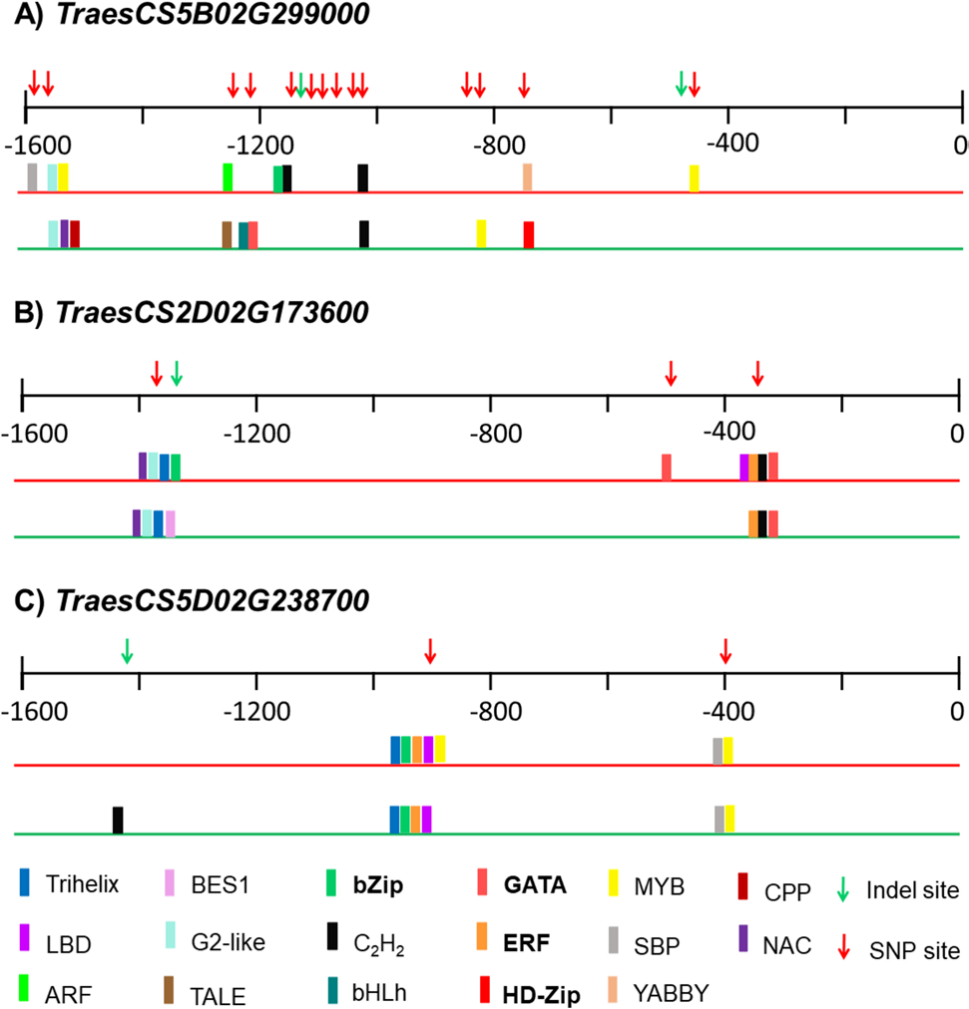
Transcription factor families with predicted binding capabilities to the polymorphic regions identified in the promoters of three calcium-binding genes (**A**, **B**, and **C**) from Syn86 (red lines) and Zentos (green lines). Families with bold letters are salt stress-responsive as indicated in Fig. 6. The binding prediction was performed with the PlantTFDB (v 5.0) database (Jin et al. 2017)

The analysis of the promoter regions of three selected genes coding for calcium-binding proteins was performed by amplicon sequencing in the two genotypes. More polymorphisms, including 14 SNPs and two deletions of single nucleotides, were detected in *TraesCS5B02G299000* (Fig. 8A). A deletion of seven nucleotides in Syn86 and three SNPs were found in *TraesCS2D02G173600* promoter (Fig. 8B), while for *TraesCS5D02G238700* one single nucleotide insertion and two SNPs were identified in Zentos (Fig. 8C). The positions of the polymorphisms in the promoter sequences are shown in the Supplementary File S1. The TF binding site analysis predicted 17 families with potential binding abilities in regions adjacent to polymorphisms. From these families, bZip and C_2_H_2_ were common in the analyzed promoters (Fig. 8). The identification of bZip, GATA, ERF, and HD-Zip binding sites in the promoters (Fig. 8) agrees with the results obtained from the transcriptomics analysis, where members from these families up-regulated under salt stress conditions were identified (Fig. 7).

### miRNA binding analysis based on SNPs identified in MACE reads

SNPs in genes coding for calcium-binding proteins were identified in the set of MACE reads previously collected from the contrasting genotypes (Duarte-Delgado et al. 2020), and miRNA binding sites were inferred in the polymorphic sites. A total of 82 SNPs were scored in 47 of the salt-responsive genes coding for calcium-binding proteins studied (Supplementary Table S4). Most of them were located in 3ʹ-UTR regions (52) followed by exons (21); six others were identified in novel 3ʹ-UTR regions predicted by Duarte-Delgado et al. (2020), and the last three in intron sequences. The surrounding sequence of eight SNPs in six genes contained potential miRNA binding sites (Table 3). From these genes, three were salt-responsive in Syn86, two in Zentos, and one in both genotypes. The possible effects of the SNP alternative allele in the miRNA affinity are shown in Table 3 and are described as the creation/loss of binding sites or increased/reduced complementarity. Five of the miRNAs found with interactions have been reported under biotic or abiotic stress stimuli in previous studies and are presented in Table 3. For instance, responsive to salt and drought stresses in wheat is reported miR171a (Alptekin et al. 2017). This miRNA might bind to the gene sequence to regulate the expression of *TraesCS5B02G428400* in Zentos.

**Table 3:** Overview of the SNPs identified in salt-responsive calcium-binding genes with potential miRNA binding in the adjacent sequence to the polymorphisms predicted with the psRNAtarget tool (Dai et al. 2018)

Gene	SNP position	Alternative allele^a^	Location in gene	Salt-responsive^b^	miRNA binding^c^	Effect alternative allele	Stimulus
*TraesCS1A02G239600*	426,881,024	**T**	3ʹ-UTR	Syn86 ↓↑	ath-miR861-5p	Loss of binding site	Nitrogen starvation (Liang et al. 2012)
	426,881,030	A					
*TraesCS1B02G251900*	444,688,564	T	3ʹ-UTR	Syn86 ↓↑	ath-miR472-5p	Loss of binding site	Insect and bacteria (Barah et al. 2013)
					ath-miR851-3p	Increased complementarity	Nitrogen starvation (Liang et al. 2012)
*TraesCS6B02G227900*	354,955,972	G	Intron	Syn86 ↓	ath-miR3933	Creation of binding site	Not reported
*TraesCS2A02G166200*	118,488,650	**G**	3ʹ-UTR	Zentos ↓↑	ath-miR5649a	Creation of binding site	Not reported
					ath-miR5649b		
*TraesCS5B02G428400*	604,067,974	G	3ʹ-UTR	Zentos ↑	tae-miR5050	Reduced complementarity	Not reported
	604,068,120	A	3ʹ-UTR		tae-miR171a	Loss of binding site	Drought (Alptekin et al. 2017),
					ath-miR171a-3p		salt (Wang et al. 2014)
*TraesCS7B02G350200*	607,529,797	**G**	Exon	Both ↓↑	ath-miR861-3p	Reduced complementarity	Not reported
					ath-miR775	Creation of binding site	Hypoxia (Moldovan et al. 2010)

^a^Bold nucleotides were found in Zentos

^b^Up-regulation (**↑**) or down-regulation (**↓**) of genes

^c^ath: *A. thaliana*, tae: *T. aestivum*

### Protein–protein interaction prediction

**Fig. 9:**
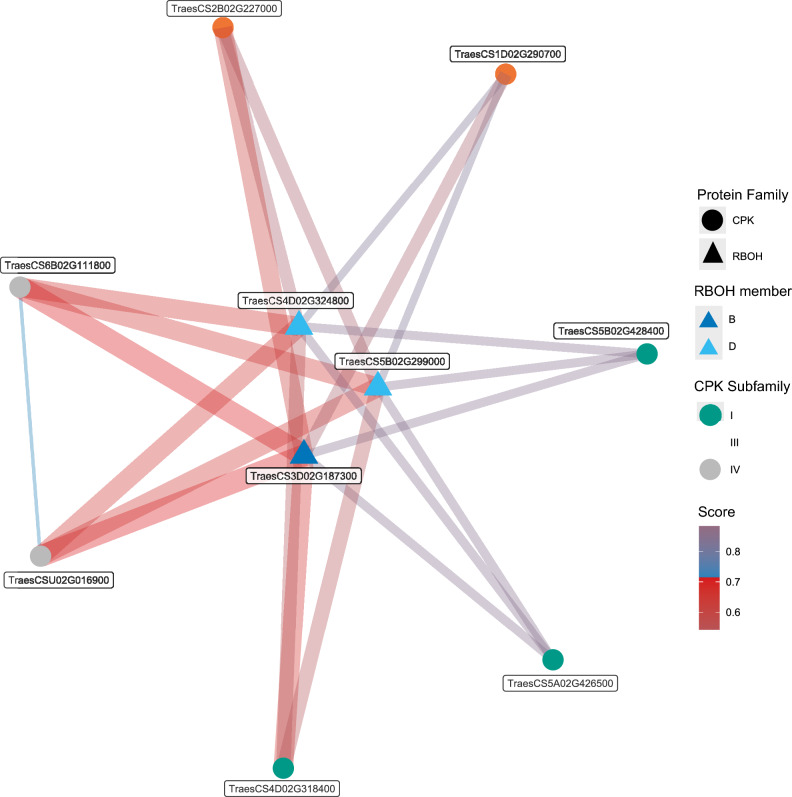
Interaction network of RBOH and CPK proteins coded by salt-responsive genes, predicted with the STRING server (Sklarzyk et al. 2023) and visualized with *ggraph* (Pedersen 2024). High prediction scores represent plausible protein–protein interactions that can be prioritized for validation studies

The network visualization revealed that the three RBOH proteins are predicted to interact with different salt-responsive CPKs with scores greater than 0.6 (Fig. 9). There are eight interactions with score values greater than 0.8 in which mostly CPKs from subgroup IV and the RBOHB ortholog are involved. The evidences that provide greater support for these high scores are the reports of putative homologs in other organisms interacting (Supplementary Table S5). Interestingly, the interactions among RBOHB and the two CPKs from subfamily IV are those with the highest scores (Fig. 9; Supplementary Table S5). The RBOHB ortholog coincided to be down-regulated at 30 min in the susceptible genotype. The protein–protein interactions with high prediction scores can be prioritized for further validation experiments.

## Discussion

### Genes involved in rapid systemic ROS production revealed by the transcriptional landscape of genes encoding calcium-binding proteins

This study depicts the transcriptional landscape for salt-responsive genes encoding calcium-binding proteins in leaves from contrasting bread genotypes. The results evidence a wide diversity of genes in the contrasting genotypes containing distinct types of architectures and coding proteins with a vast array of functional domains. This diversity influenced the triggering of specific stress responses in systemic tissues during the osmotic phase (Gilroy et al. 2016). The domain analysis of the calcium-binding proteins coded by salt-responsive genes indicated that there are different types of them in the two genotypes as Zentos was enriched with genes annotated to encode proteins with EF-hand domain, while Syn86 displayed a prevalence of the non-EF-hand category. The variation in the categories of stress-responsive genes involved in Ca^2+^-dependent networks on the contrasting genotypes represents a differential execution of signal transductions and suggests a particular calcium signature that can be related either to tolerance or susceptibility responses during the osmotic phase (Sanyal et al. 2019; Mohanta et al. 2019).

Additional genes coding proteins with non-EF-hand domain related to the susceptibility response from Syn86 were highlighted. These categories of genes complemented the transcripts associated to proteins from the OEC from the photosystem II involved in the photosynthesis reduction from the susceptible genotype that were previously discussed (Duarte-Delgado et al. 2020). For instance, annexins are plasma membrane proteins activated by ROS to function as Ca^2+^ channels (Moinoddini et al. 2023). The observed down-regulation of annexins could alter the transport of Ca^2+^ and affect the [Ca^2+^] cytosolic signals to be decoded by intracellular sensors (Liao et al. 2017; Sanyal et al. 2019). The up-regulation of CNX/CRT transcripts can be related to the greater oxidative stress response observed at the transcriptional level in this genotype (Duarte-Delgado et al. 2020). This calcium-binding protein type is involved in the pathway from the endoplasmic reticulum to refold and degrade stress-damaged proteins (Garg et al. 2015). We also identified the up-regulation of phosphoglycolate phosphatases, which corresponded to the EF-hand group. This transcriptional response from the susceptible genotype might be linked to the activation of the photorespiration metabolism to alleviate photoinhibition for stress protection (Wingler et al. 2000; Schwarte and Bauwe 2007). Thus, we could identify further transcriptional responses involved in calcium signaling related to susceptibility responses.

Genes coding proteins with EF-hand domains that detect transient increases of cytosolic Ca^2+^ and activate proper stress responses (Sanyal et al. 2019), were further studied through a phylogenetic analysis. Wheat CPKs and RBOHs showed clear orthologous relationships with the corresponding Arabidopsis proteins. The lack of clear orthologs for CaM and CML indicates greater sequence divergence along evolutionary time, and therefore gene function might be less conserved in distant taxa (Eisman and Kaufman 2013). The sequence similarity relatedness among the two species contributed to infer some stress response pathways in which the salt-responsive genes from Syn86 and Zentos might be involved.

For instance, the RBOH family members produce localized signaling ROS bursts and were of special interest in our study due to their involvement in rapid systemic stress responses (Choudhury et al. 2017; Chapman et al. 2019). The clustering with high statistical support of two wheat proteins with RBOHD from Arabidopsis is consistent with its reported role in salt stress response, systemic acquired acclimation by abiotic stress, and regulation of ABA-mediated stomatal closure (Chapman et al. 2019; Kaya et al. 2019). The RT-qPCR analysis in Zentos revealed the up-regulation of both RBOH genes in leaves at 8 min ASE, which coincided with a faster calcium and ROS signaling that is proposed as a crucial mechanism to trigger salt tolerance (Ismail et al. 2014). The higher expression levels of *TraesCS5B02G299000* might be responsible for tolerance-triggering mechanisms in Zentos. Contrary to our study, Arabidopsis RBOHD was up-regulated exclusively in roots starting at 3 h ASE, and afterward RBOHE and RBOHF were expressed in leaves (Suzuki et al. 2011). These results suggest that a different RBOH member might regulate the onset of stress sensing in wheat roots at an earlier time point.

The kinase-substrate interactions from CPK members from subgroup I and RBOHD in Arabidopsis are reported to regulate TFs under abiotic stresses (reviewed in Yip-Delormel and Boudsocq 2019). In our study on the opposite, the interactions of subgroup IV CPKs and the RBOHB ortholog are predicted with higher scores based on interactions reported for other organisms. The down-regulation of this ortholog in Syn86 can provide some insights on molecular mechanisms influencing the susceptibility response. For instance, the rice ortholog OsRBOHB has been identified as a crucial player in drought and salt tolerance (Shi et al. 2020). These results suggest proteins involved in a novel signaling pathway for bread wheat during salt stress response. Biochemical assays can be integrated to validate the prioritized phosphorylation-mediated putative interactions. The combination of a yeast two-hybrid assay with bimolecular fluorescence complementation was proposed to confirm a CaM-kinase interaction in bread wheat (Li et al. 2022).

### Genes coding for calcium-binding proteins are predominantly co-expressed with WRKY and AP2/ERF members

This study suggests an important role of AP2/ERF and WRKY families during the osmotic phase response, as members from these families were the most abundant among the up-regulated TFs. According to studies in Arabidopsis, the simultaneous expression of AP2/ERF family members and genes coding proteins with EF-hand domain is intertwined. For instance, RBOHD expression is activated by the binding of ERF74 in the promoter region (Xie et al. 2019) and the transcription of some DREB genes from the AP2/ERF family is regulated by calmodulin-related pathways (Jan et al. 2017). Genes corresponding to the DREB subfamily showed greater relative expression values in the tolerant genotype, which can be related to the predominant activation of the ABA-independent signal transduction pathway during stress (Erpen et al. 2018; Li et al. 2019).

The fast expression of WRKY genes identified during the osmotic phase can contribute to the transcriptional activation of adaptation mechanisms related to ABA signaling and the production of secondary metabolites with relevance on stress responses (Banerjee and Roychoudhury 2015; Phukan et al. 2016). The co-expression of WRKY and GATA TFs identified in our study agrees with the transcripts identified in Arabidopsis due to ROS/Ca^2+^-dependent signaling activated after few minutes of light excess (Zandalinas et al. 2019). The proteins coded by the three salt-responsive WRKY genes from Group IId detected in bread wheat are potential interactors with EF-hand proteins. In *A. thaliana* a CaM-binding domain has been identified and validated for this group of WRKY proteins (Banerjee and Roychoudhury 2015; Seifikalhor et al. 2019). The STRING database (Szklarczyk et al 2023) did not predict interactions for this subgroup of WRKY proteins and therefore alternative methods might be utilized to prioritize putative protein–protein interactions to implement biochemical assays. The co-expression analysis suggests a fine-tuned interplay among genes coding for calcium-binding proteins and specific TF families in pathways to trigger salt stress responses. Our results pinpoint additional genes in these pathways to pursue RT-qPCR in leaves and roots to gain a deeper understanding on their role during immediate osmotic stress response.

### Genetic variation with potential influence on expression levels of genes annotated to encode proteins with calcium-binding domain

Several TF families showed potential binding sites adjacent to the identified polymorphisms in the promoter regions of the genes coding for proteins with calcium-binding domain studied. Among them, the families GATA, bZip, HD-Zip, and ERF/AP2 were identified as salt-responsive in the comparative transcriptome analysis, highlighting them as putative regulators of the differential expression of the genes coding for proteins with calcium-binding domain in the contrasting genotypes. These regulations of expression levels are supported by studies such as the one reporting the binding of a bZip member to the promoter of a gene encoding a CaM protein in Arabidopsis (Reddy et al. 2011). The identification of *cis*-regulatory polymorphisms in genes annotated to encode proteins with calcium-binding domain with affinity to ERF/AP2 and GATA families may underlie the co-expression of both gene categories that was discussed in the previous section. Interestingly, the polymorphisms detected in the promoter from *TraesCS2D02G173600*, a candidate gene identified within a salt stress response QTL (Dadshani 2018), can explain the up-regulation of this gene coding a CML protein in Zentos (Duarte-Delgado et al. 2020) and the greater tolerance to salt stress found in this genotype. A transient expression assay in a heterologous system under stress conditions can be used to assess the activity of the promoter alleles, as proposed by Muzammil et al. (2018) for barley to validate polymorphisms associated to drought response.

The analysis of MACE reads led to the identification of SNPs to infer putative miRNA binding sites in the transcripts. Few SNPs were scored in intron regions and extended gene models described by Duarte-Delgado et al. (2020). The reads mapping to intronic regions can be due to imprecise annotations and they can be a proxy of alternative transcription and splicing events (Gaidatzis et al. 2015). Plants can respond to environmental stresses by altering gene expression through the activity of miRNAs (Khraiwesh et al. 2012; Asefpour Vakilian 2020). The potential interaction of the CPK transcript *TraesCS5B02G428400* and tae-miR171a agrees with the report of this miRNA as salt- and drought-responsive in wheat and other Triticeae species (Wang et al. 2014; Alptekin et al. 2017). This transcript is stress-responsive for a short period, which can be related to tissue- and time-specific expression of miRNAs after salt stress exposure (Asefpour Vakilian 2020). A fluorescent-based RNA electrophoretic mobility shift assay (FREMSA) and a dual-luciferase reporter system can validate the miRNA-mRNA interactions and the effect of the 3ʹ-UTR polymorphisms (Guo et al. 2020).

## Conclusions

The comprehensive analysis of the transcriptional landscape of genes coding for proteins with calcium-binding domain at the initial minutes and hours of the osmotic phase during the salt stress response revealed differences underlying the contrasting stress responses of the studied genotypes. The non-EF-hand category was specific for the susceptibility response, as revealed by the regulation of transcripts related to oxidative stress protection and oxidative stress damage repair. On the other side, Zentos, the tolerant genotype was characterized by a faster and higher up-regulation of genes annotated to encode proteins with EF-hand domain and TF members. The transcription of these genes might be involved in signaling pathways to trigger and increase salt tolerance, such as the early expression of RBOHD orthologs involved in ROS production. This study provides insights into the interplay of calcium-binding proteins, WRKY, and AP2/ERF TF families in signaling pathways at the start of the osmotic phase to affect the expression of several downstream genes. Furthermore, the identification of natural variation in *cis-*regulatory sequences provides insights into mechanisms related to the differential expression levels observed among the contrasting genotypes, which can support the effect of QTL regions related to stress response.

## Supplementary Information

Below is the link to the electronic supplementary material.

Supplementary file1 (DOCX 1203 KB)

Supplementary file2 (DOCX 592 KB)

Supplementary file3 (DOCX 619 KB)

Supplementary file4 (XLSX 12 KB)

Supplementary file5 (XLSX 18 KB)

Supplementary file6 (XLSX 22 KB)

Supplementary file7 (DOCX 30 KB)

Supplementary file8 (XLSX 24 KB)

Supplementary file9 (DOCX 17 KB)

## Data Availability

The data supporting the results of this article are included within the article and the provided supplementary files.
