# Supplementary material for: Expression interplay of genes coding for calcium-binding proteins and transcription factors during the osmotic phase provides insights on salt stress response mechanisms in bread wheat
Source: Plant Mol Biol. 2024 Nov 1;114(6):119. doi: 10.1007/s11103-024-01523-z (PMC11530504; doi:10.1007/s11103-024-01523-z)

**Figure S1.** Melting curves of some PCR products from the amplification of studied RBOH genes.

1. ***TraesCS4D02G324800***


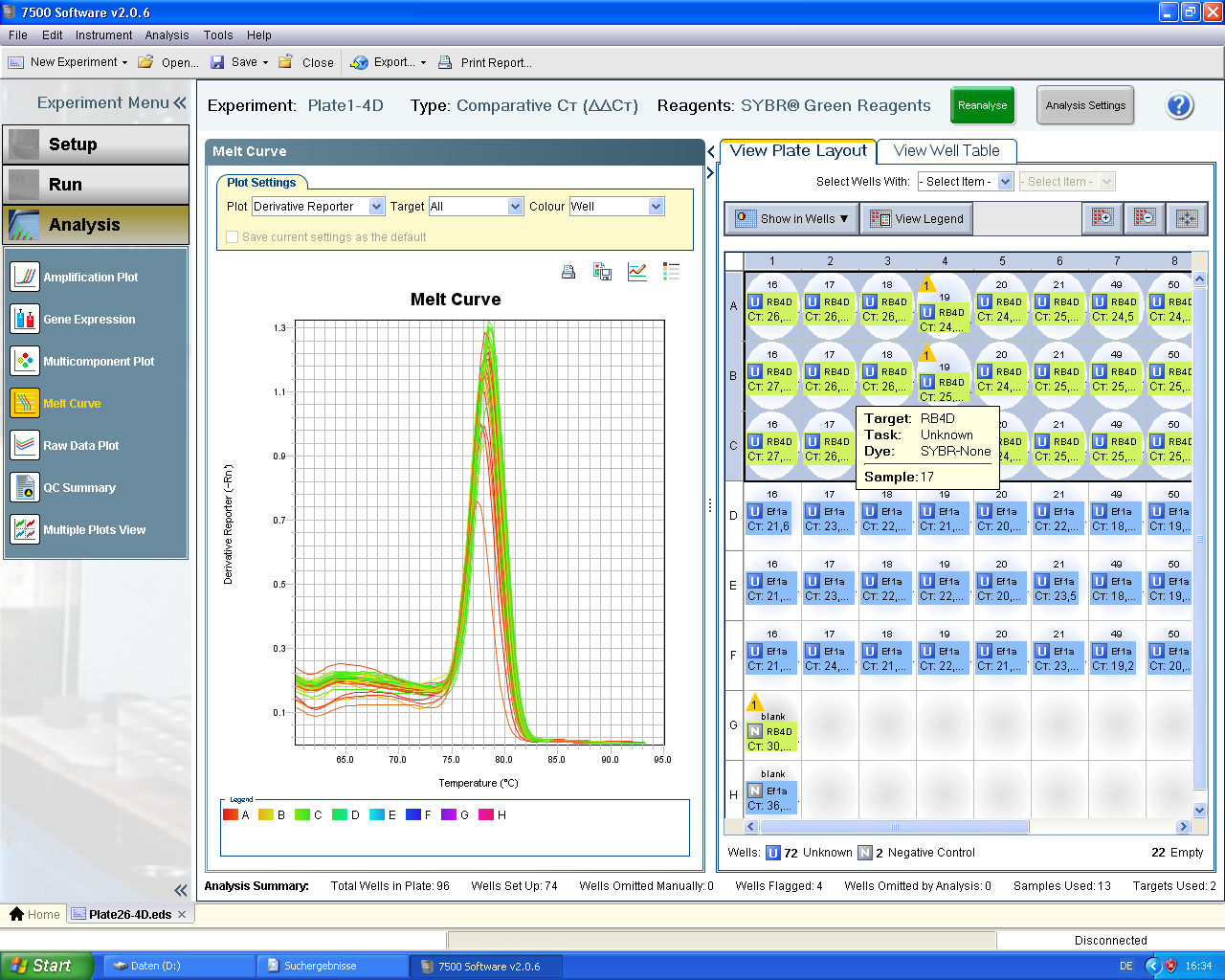


1. ***TraesCS5B02G299000***


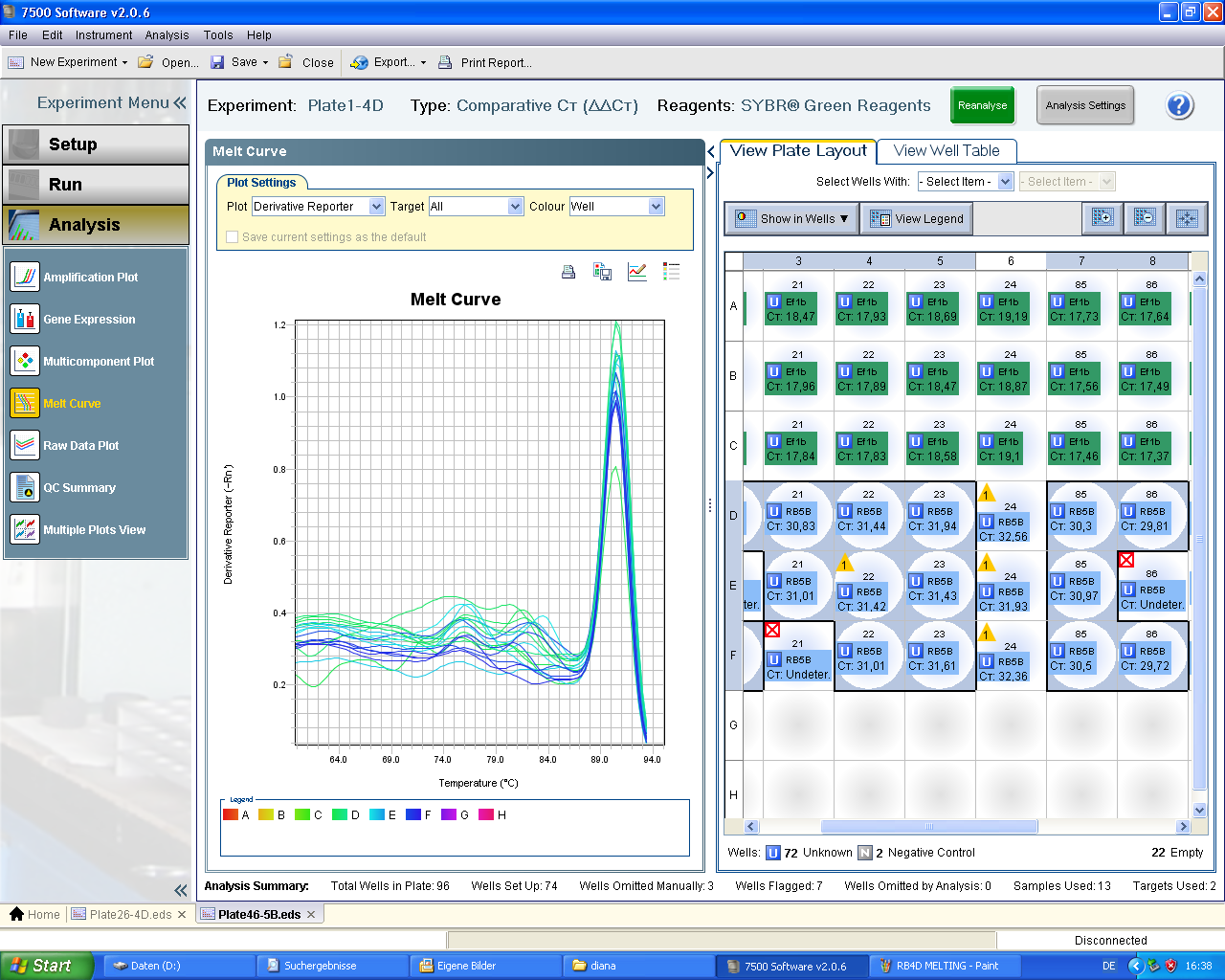

Supplement: Supplementary file 1 — Supplementary file1 (DOCX 1203 KB) [file 11103_2024_1523_MOESM1_ESM.docx]
