# Supplementary material for: Expression interplay of genes coding for calcium-binding proteins and transcription factors during the osmotic phase provides insights on salt stress response mechanisms in bread wheat
Source: Plant Mol Biol. 2024 Nov 1;114(6):119. doi: 10.1007/s11103-024-01523-z (PMC11530504; doi:10.1007/s11103-024-01523-z)
Supplement: Supplementary file 2 — Supplementary file2 (DOCX 592 KB) [file 11103_2024_1523_MOESM2_ESM.docx]

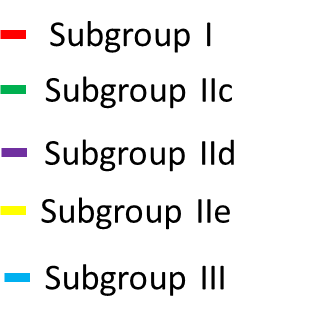

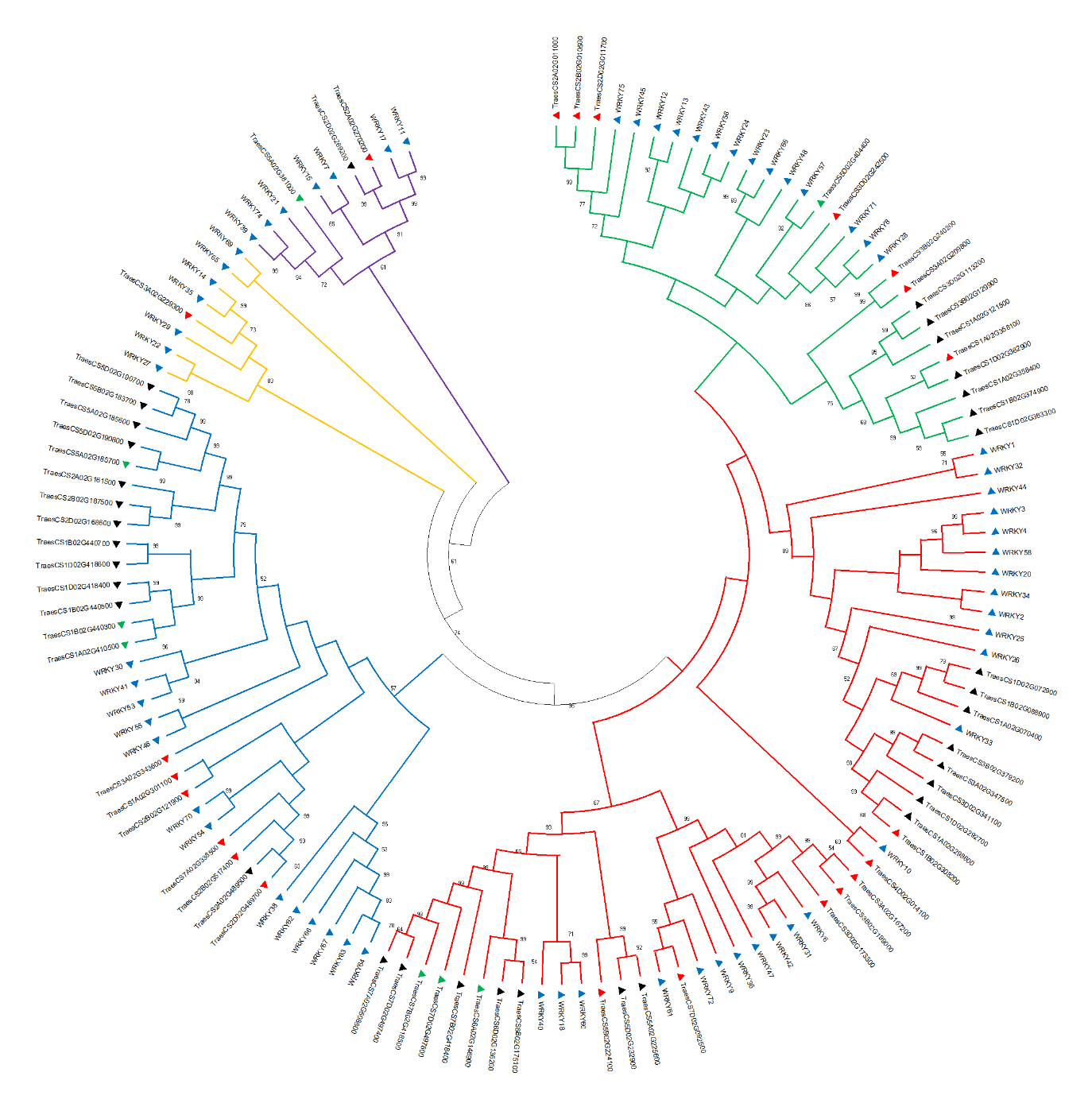

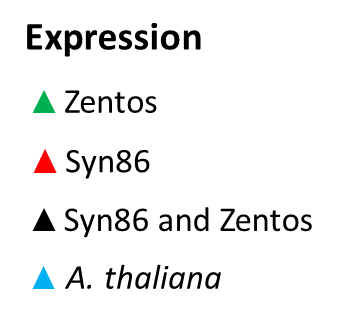


**Figure S2.** Dendrogram of WRKYs amino acid sequences from *Arabidopsis thaliana* and coded by the corresponding salt-responsive genes from Zentos and Syn86 indicated by the colored triangles. The consensus phylogenetic tree was constructed with MEGA X (Kumar et al*.* 2018) using the Neighbour-Joining method and through a bootstrap analysis of 3000 replicates. The subgroups are indicated by colors in the branches.
