# Supplementary material for: Expression interplay of genes coding for calcium-binding proteins and transcription factors during the osmotic phase provides insights on salt stress response mechanisms in bread wheat
Source: Plant Mol Biol. 2024 Nov 1;114(6):119. doi: 10.1007/s11103-024-01523-z (PMC11530504; doi:10.1007/s11103-024-01523-z)
Supplement: Supplementary file 3 — Supplementary file3 (DOCX 619 KB) [file 11103_2024_1523_MOESM3_ESM.docx]

**
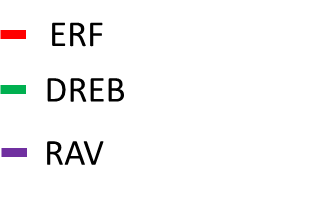
**
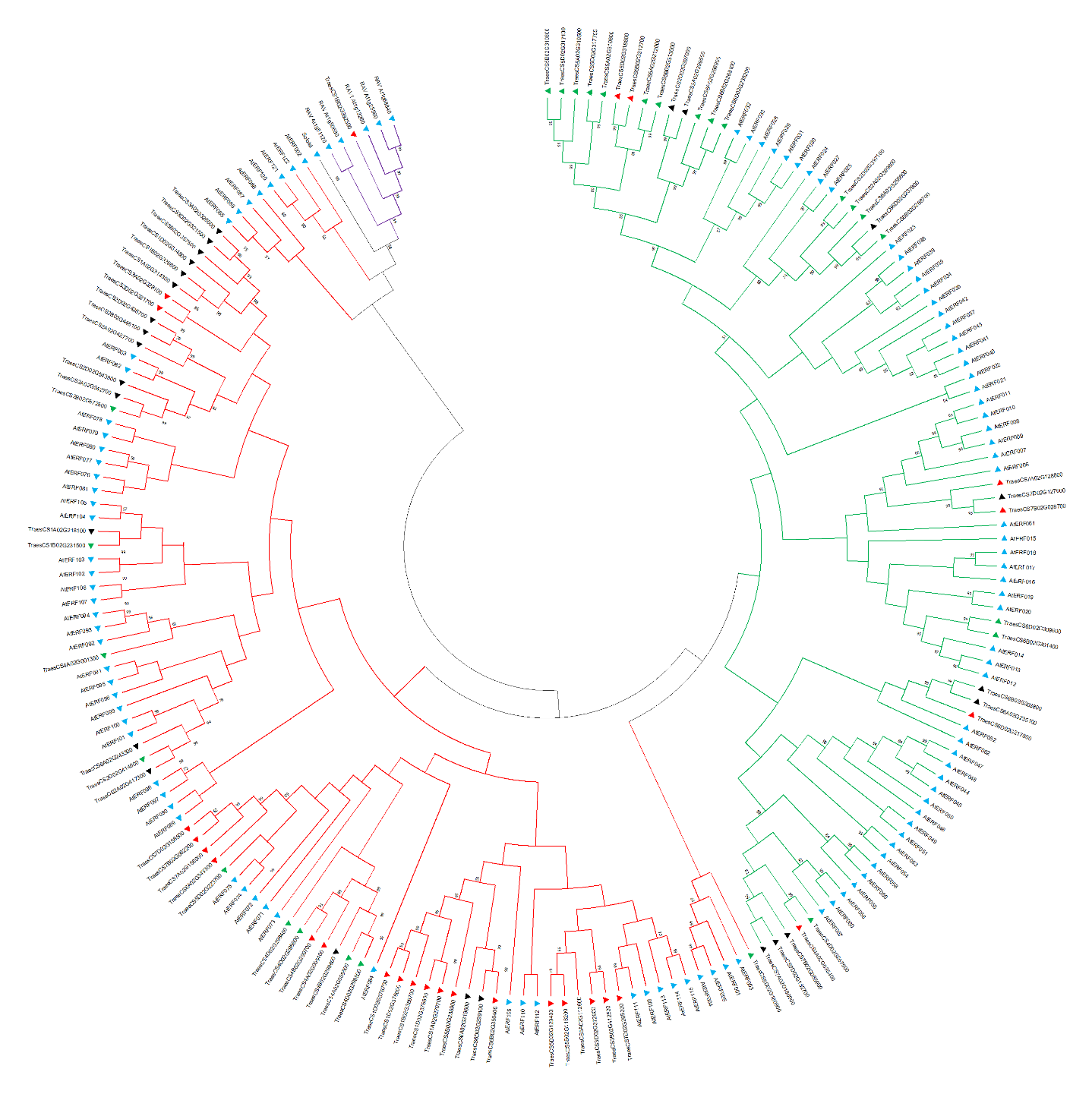

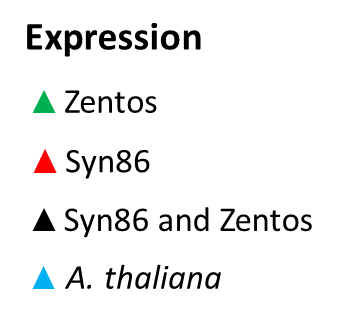


**Figure S3.** Dendrogram of amino acid sequences from ERF (red), DREB (green), and RAV (purple) subfamilies from *Arabidopsis thaliana* and coded by the corresponding salt-responsive genes from Zentos and Syn86 indicated by the colored triangles. The consensus phylogenetic tree was constructed with MEGA X (Kumar et al*.* 2018) using the Neighbour-Joining method and through a bootstrap analysis of 3000 replicates.
