# Supplementary material for: Expression interplay of genes coding for calcium-binding proteins and transcription factors during the osmotic phase provides insights on salt stress response mechanisms in bread wheat
Source: Plant Mol Biol. 2024 Nov 1;114(6):119. doi: 10.1007/s11103-024-01523-z (PMC11530504; doi:10.1007/s11103-024-01523-z)
Supplement: Supplementary file 7 — Supplementary file7 (DOCX 30 KB) [file 11103_2024_1523_MOESM7_ESM.docx]

**Supplementary Table S4.** SNPs identified in salt-responsive genes with calcium-binding domain. The reference allele is represented by 0 and the alternative allele by 1.



| **Gene** | **Position** | **Alternative** | **Syn86** | **Zentos** | **SNP location** | **Salt responsive** |
| --- | --- | --- | --- | --- | --- | --- |
|  |  | **allele** |  |  | **in gene** |  |
| TraesCS1A02G239600 | 426881024 | T | 0/0 | 1/1 | UTR | Syn86 |
|  | 426881030 | A | 1/1 | 0/0 | UTR |  |
| TraesCS1B02G251900 | 444688564 | T | 1/1 | 0/0 | UTR | Syn86 |
| TraesCS1B02G370900 | 600605115 | A | 1/1 | 0/0 | UTR | Syn86 |
| TraesCS2A02G215000 | 202042541 | C | 0/0 | 1/1 | UTR | Syn86 |
| TraesCS2A02G545600 | 754357893 | A | 1/1 | 0/0 | UTR | Syn86 |
| TraesCS2B02G113800 | 77170854 | C | 0/0 | 1/1 | UTR | Syn86 |
|  | 77170975 | A | 1/1 | 0/0 | UTR |  |
|  | 77170544 | C | 0/0 | 1/1 | exon |  |
|  | 77170646 | T | 0/0 | 1/1 | exon |  |
| TraesCS2B02G376700 | 539651338 | G | 0/0 | 1/1 | UTR | Syn86 |
| TraesCS2B02G576100 | 765069399 | T | 0/0 | 1/1 | UTR | Syn86 |
|  | 765069719 | G | 1/1 | 0/0 | exon |  |
| TraesCS3A02G335000 | 581276075 | C | 1/1 | 0/0 | UTR | Syn86 |
| TraesCS3A02G454900 | 692961935 | G | 1/1 | 0/0 | UTR | Syn86 |
| TraesCS3B02G362600 | 574267558 | G | 1/1 | 0/0 | UTR | Syn86 |
|  | 574267731 | G | 1/1 | 0/0 | UTR |  |
| TraesCS3D02G499700 | 589417216 | C | 1/1 | 0/0 | UTR | Syn86 |
| TraesCS4A02G310100 | 603038176 | G | 1/1 | 0/0 | unpredicted UTR | Syn86 |
| TraesCS4A02G310600 | 603372837 | G | 1/1 | 0/0 | UTR | Syn86 |
|  | 603372858 | T | 1/1 | 0/0 | exon |  |
|  | 603372757 | T | 1/1 | 0/0 | UTR |  |
| TraesCS4D02G003600 | 1415905 | G | 1/1 | 0/0 | UTR | Syn86 |
| TraesCS4D02G174400 | 302399810 | C | 1/1 | 0/0 | intron | Syn86 |
| TraesCS5A02G261200 | 475269281 | T | 1/1 | 0/0 | exon | Syn86 |
| TraesCS5A02G461500 | 641733968 | A | 1/1 | 0/0 | UTR | Syn86 |
| TraesCS5B02G063600 | 71521277 | A | 1/1 | 0/0 | unpredicted UTR | Syn86 |
| TraesCS5B02G247400 | 429093157 | A | 1/1 | 0/0 | unpredicted UTR | Syn86 |
| TraesCS5B02G462600 | 638124329 | C | 0/0 | 1/1 | UTR | Syn86 |
|  | 638124438 | G | 1/1 | 0/0 | exon |  |
| TraesCS5D02G474200 | 513978818 | C | 1/1 | 0/0 | exon | Syn86 |
| TraesCS6A02G101800 | 70573375 | C | 0/0 | 1/1 | UTR | Syn86 |
|  | 70573390 | T | 0/0 | 1/1 | UTR |  |
| TraesCS6B02G037600 | 22105419 | A | 1/1 | 0/0 | unpredicted UTR | Syn86 |
|  | 22105468 | G | 1/1 | 0/0 | unpredicted UTR |  |
|  | 22105558 | G | 1/1 | 0/0 | unpredicted UTR |  |
| TraesCS6B02G129800 | 126220744 | A | 0/0 | 1/1 | exon | Syn86 |
|  | 126220872 | C | 0/0 | 1/1 | UTR |  |
| TraesCS6B02G227900 | 354955972 | G | 1/1 | 0/0 | intron | Syn86 |
| TraesCS6D02G090400 | 56277741 | A | 1/1 | 0/0 | UTR | Syn86 |
| TraesCS7A02G245300 | 223472910 | A | 0/0 | 1/1 | UTR | Syn86 |
|  | 223472964 | C | 1/1 | 0/0 | UTR |  |
| TraesCS7A02G248600 | 230566681 | T | 1/1 | 0/0 | exon | Syn86 |
| TraesCS7A02G483700 | 675232270 | C | 1/1 | 0/0 | exon | Syn86 |
|  | 675232271 | G | 1/1 | 0/0 | exon |  |
| TraesCS3A02G038300 | 20589880 | A | 0/0 | 1/1 | UTR | Zentos |
|  | 20589932 | G | 0/0 | 1/1 | UTR |  |
|  | 20590267 | A | 0/0 | 1/1 | exon |  |
|  | 20590404 | G | 0/0 | 1/1 | exon |  |
|  | 20590405 | C | 0/0 | 1/1 | exon |  |
| TraesCS2A02G166200 | 118488650 | G | 0/0 | 1/1 | UTR | Zentos |
|  | 118488839 | T | 1/1 | 0/0 | UTR |  |
| TraesCS3D02G041400 | 15937155 | T | 1/1 | 0/0 | UTR | Zentos |
| TraesCS5A02G426500 | 611567227 | A | 1/1 | 0/0 | intron | Zentos |
|  | 611567555 | A | 1/1 | 0/0 | exon |  |
| TraesCS3D02G041500 | 15943904 | T | 1/1 | 0/0 | UTR | Zentos |
|  | 15943959 | C | 1/1 | 0/0 | UTR |  |
| TraesCS5B02G428400 | 604067961 | T | 1/1 | 0/0 | UTR | Zentos |
|  | 604067974 | G | 1/1 | 0/0 | UTR |  |
|  | 604068106 | G | 1/1 | 0/0 | UTR |  |
|  | 604068120 | A | 1/1 | 0/0 | UTR |  |
|  | 604068263 | G | 1/1 | 0/0 | UTR |  |
| TraesCS2B02G181800 | 156606159 | C | 1/1 | 0/0 | UTR | Zentos |
|  | 156606173 | G | 1/1 | 0/0 | UTR |  |
|  | 156606246 | G | 1/1 | 0/0 | UTR |  |
|  | 156606294 | C | 1/1 | 0/0 | UTR |  |
| TraesCS7D02G439800 | 559393803 | G | 1/1 | 0/0 | UTR | Both |
| TraesCS2A02G156400 | 103139824 | T | 0/0 | 1/1 | UTR | Both |
|  | 103139852 | A | 0/0 | 1/1 | UTR |  |
|  | 103139965 | G | 0/0 | 1/1 | UTR |  |
|  | 103140100 | C | 0/0 | 1/1 | exon |  |
| TraesCS2B02G182000 | 157040899 | A | 1/1 | 0/0 | UTR | Both |
| TraesCS4A02G123100 | 153779965 | A | 1/1 | 0/0 | exon | Both |
|  | 153780078 | G | 1/1 | 0/0 | UTR |  |
| TraesCS4B02G121300 | 142928707 | C | 1/1 | 0/0 | UTR | Both |
| TraesCS5A02G229000 | 444891521 | T | 0/0 | 1/1 | exon | Both |
|  | 444891771 | G | 1/1 | 0/0 | UTR |  |
| TraesCS5B02G227700 | 404184046 | C | 0/0 | 1/1 | exon | Both |
|  | 404184096 | A | 0/0 | 1/1 | UTR |  |
| TraesCS5B02G396400 | 573226206 | T | 1/1 | 0/0 | UTR | Both |
| TraesCS7A02G450400 | 644104500 | T | 1/1 | 0/0 | exon | Both |
| TraesCS7B02G350200 | 607529797 | G | 0/0 | 1/1 | exon | Both |
