# Supplementary material for: Expression interplay of genes coding for calcium-binding proteins and transcription factors during the osmotic phase provides insights on salt stress response mechanisms in bread wheat
Source: Plant Mol Biol. 2024 Nov 1;114(6):119. doi: 10.1007/s11103-024-01523-z (PMC11530504; doi:10.1007/s11103-024-01523-z)
Supplement: Supplementary file 9 — Supplementary file9 (DOCX 17 KB) [file 11103_2024_1523_MOESM9_ESM.docx]

**Supplementary File S1:** Polymorphisms identified in the promoter sequences from three calcium-binding genes. The alternative allele color is red for Syn86 and green for Zentos

Promotor *TraesCS5B02G299000*

chr5B: 482286000..482288300

GGATTTGGAATTGATGAATGTTGCTCTGTTAGCTAAATGTCTTTAGAAACTTTTTAATGAACATGGTCTCCGGCAGGATTTTCTATGCGCAAAATATGTTGCTAGTTCTACTCTTGGTCAGGTTCAACTTAGATGAGGGGACTCACATTTTAGGCAGGGTTTGCTAAAAATTAAAGGGCTCTTCCTGTCTAGATTCTGGGAAGATAGATGGCTGGGAAATGAAAGCCTATCTCAAACCTTCCCAAGACTTTATAGTATTTCTCTTGATCATAACATTACTGTACATGATGTTTTTACTGGTTGTCTCTCCAAACTTTCTTTTCGGAGGGCCTTGGTTGGGGAGAAGGAGATCCTTTGGTCTAAATTACTAGACACATGTGCCCCTGTTACTTTGTCTGAATCTGAAGATAAATTGTCTTGGATTATAGACAAATCTGGAGTATACCGGTTTAAAATCCTTCTACTCGGCTATGTAGGTTGGATGTAAAGT(C/T)CCTTACA(G/A)ATTTTCGTGGAAAGTTAAAATTCCTTCTAGAATCAGAATGTTCATATGGTTAGTGCTGAGGAAAAGCATATTGACCAAAGATGTTCTACTATATTTGTTTTGTGGACAAAAAGAAACGATTGATCATCTCTTCTTTGAATGTCCCCTTGCAAGTTAAATTTGGTGTGTGATGAAATGTAGTTTTGGTGTTGATTGTTCTTTTATGGACACAGAAGTTTGTTTTACTGTTTGGCTTAAAAACTTACCTACCCAGAGAAGGAAGCTATACTCAGTGGGAGTGGCAGCTGTTCCTGGGGAATTC(G/T)GACAGCCAGGAATCTAGCATGCTTTGAGCTTAAATGGCC(A/T)TCTGATCCATTTGTTGTGATGTTTAGGGTAGCCCACTGGATTGAGTTCACTACAG(A/G)TGAAGGAGGACGCAAAGGTGG(C/)(G/A)CTCTGACAGGGC(A/G)CAAAAATTCTG(A/G)AACGAGTCGCAACTAATG(T/C)GTTCCAAGCCAGTG(C/T)CATGGATCCCTCGACTGGAAGATGGGGGTTGAGTCTGGAGTGCACGGAGAGATGTTGTGTAATAACAAAAACCGGATTACTTTGAGTTTTGTTTTGTTCTAGCTTGAGTTAACTTTGGTTGGTCAGGTCCACCTAGTCTATACTTGTAACCAAGGATTTGGTCACCGAGC(G/A)AAAAAATCC(G/A)TTACCGCCCGGTAACCACGAATCTTGGTACCGCACGAGAAATCTCATTACCAGGCAAAAAAAAAAAAAACTGATTTTGTGAATTTTGAATGAATTTTATTTGAATCTGGAATCCATTA(A/T)AAAAATTGTTGCTAACCTCCCGGCGTTTTCCAGTTACCTTGGTGTAGCTGAAAGTCTCAGTGCTACACCAATCTTTTCTTTTCTTTCGGAATCCAAATCCTTGCTTCCAACAGGTTTTTCTTTTTGAGGGAAAGCAACTTTAGTTGACGCGGAATGACAACTTTAATTGTTAAAATATGACAATTTTATCCTAGCTCGTTTTCCTGTCAGAAAATTGTCATGTTTGCCAACTACGCGTGAACTAAAGTTGCCATAAAAA(A/)CGTT(C/T)AGGTTGTCATGCATAAAATCCGGGCGTTCGGGATTTATTATTTTCGTTGTTTTTTACTACACCAACTGCACAATTTTATTTTTGTTGCAAACTAAAAGTTTGCGTGTGCTGATTTCTTTCATTTTGAGTAAGAGTATCAGCGCTGCTGTCGCTGTATACATGCGCCAAAAAGGGAACAATTCTCGTCGCACCTATCCATCGAGAGGAACGGCGCCGTTCGTGCTCAATAAATGCTAAGCAGCAATTAGAGACCTGTCCCAACTTGCAACCCCTGGCGCGTACGTAAACCGCGTGCATGCATGCGGCCGTTGGAGCAAGCGCCGCGGTCGCGGTCAAGCGACCTTTTGTTCTGACCAACAGGGCACGCCGCCTTCGTAGGTCGTAGTAGGTGAGTTCCCTGGCTGAAAGGGTCGCCGGAGAATTTCACCAGACTTCATTTTTTCGACATAAATTTCGCGAGACTTTGCGCCACGCTGAAACAGTCCCTCCCTCCCGGCG(G/A)G(C/T)TGCTATATTAGGACGAGCAGCGTTGAACTGTTTGCTTAGCACTGCTAGCTCCGGTGCGTGATCTCATCACCATCTCCTCCTCCACCTACTACGTGAGCCTAATCCTCCGGCCGGCCGCGACTACGTCCATTAAATCCTGCTTCCGCGGCGCGCTCTGGCTGCGCATCTTGGCTGGCCGGTTGACTTTGTTGGGGACGGGCGAGCTCTAGCTGGAGCCGGCGGCCGGCCATGCATAACTACGGAGGCGGCGGCGGGG

Promotor *TraesCS2D02G173600*

chr2D:117 219 829..117 222 419

TGAGATGAAATAAAATTGGTGAATCCCGACAATGCTTGGGACTCATAAGTAATTTCCAATGGAGCATAAATACTATGTTGTAAAAGGTCTACAAGGTCAATGTGACTCCAAAAGAAATATGTAAAGGTGTAAAGCGCGACTTGAATCGAAAGATTTTGTGCAAAGAGAATGAACAAATTACAATGAGTGTTAGTGTCACATCATGACCGACATCATGATCTGAGTTACATCAGATGAATGTAAAGAACACAAGGGATACTGCTTCAAGAAATTTTTTATGGACTAGAGCAAGTCTCTAGATAGTTGCATTTAAAGTTTAAAGAATCAGATGATATTTTGGGTTAAAGAAAATGGAAGGACAATTGTATTCATGCAAAGTTATAGAATGGGAAATTCATTTCCCAATCTTGTGCATGTGGATGACGTCCTACTTGCTAGTGGTGATGTCAATCTACTTCAGGAGGAGAAAAGAAGTTCTTGTCCTCAAAGTTCGAAATTGCATTTCTCAGAGTGTCTCTCGTTATAAATATCGAGATTCATCAAGAAAAGAATAAAATAGGGTATTAGGAATGTCACATGGACATGCTAACAAAGGTCTCTAAAGTATGGATGCGAGAAAACCTACGCCTGTTCTTATAGTCAAGGGTAATGGAACTGGAAACTATGGTGTTCCAAAAGTTGATGAGAAAAGATTGAAAACGGATATGGTACCATATGCTTCAGCTGTTGGAAGCTCACTATATTACC**[C/T]**TGA**[CACAATT/]**CACGTATCCGGGTTGTTTTGGCAATATCCAGTCCATATATAGATCACTGGAATGGAGTCAAAGATATCGGCCTCGTGCTGAAAGAAATAAGTGCTCTCAAAAGATTGTGAGTACAAAGACAGGACTTGTGAAATGTATAGCGAAATCCACAATTGTCGCTAACTTTCATACTTGGAGATTTTTGTGTGGAAAAGCTCCAAAGAATGAAACAATTATCATCAATGTGATGCAAAGATATTTTATAGCTTGATATGAGGCTGAGGGACAGGCAAAATGGTTAAGGAGACCTGTACCCGGAGTTAATAATGGTTGACAACAGCGATAACCATTTTAAGTTGTTCGCTCCTATGACAACGAGTCAAGTGTTGATGCCAAACAAACTGACACAGAGTTATGCGTTGTAAAGGAGAAAGTCCGGAATTATGTAGAAATGCTTGAAGCATAAAAGCAACAGACAAGTGTTTGCAGATCTGCTTATTAAAGGCTTACCGCCCAGTGTGTTCGGAGAACACACAGTCGACATGGGTTTTATGGTATAGTCTAAGATTTCCGGACAATAAAAGGGCCCAAGGTTAAAGAATTTGTTTCAAAACAGAAAGGTACGTTGTGGCTGTCTGATTCTATCGGCAATTGAGCTGTGACGATGAAACATGTTCTATGTATTGATCTATTATGAAACGAGTAAAGTAAAAGTATAAGGTCAAAAGTAAAAGTTGAGATCAAGGGGGAGAATGTTAGGATGATCTCCACCGTGTGGGCCCAACGGCCCACCGGGCCCTTAGATCT**[A/G]**CGCCCTGATTGAGGGCGCGGTTGACGGGCCCCTGTCACCTGCACTATATAAAGAGGTGGGGGCCGGCGGCTCGCATCACGAGGTTCGTCGCGACGCCGTACACCCCACCTACATCCCCTACCGATCTAGAGTTAGTGCAGTGCTGACGGGAAGCACCGCCACCGCTACTCTGCCATTACCGGCCACCGTCACCATGGCCGGCA**[T/C]**CGGGAGCTCCTCGAGCCACAAGAAGAAGGTAATACCTACCACCACTGTTGCCGGAATCCTAGCCTAACCGATCCACAGAATCTATCACCTTCAACATCTTAGCCCAACTGTTAGCTCTGAATGAATGGATGCAGGCATGGTTAACCCTGCATATTTTCTCTGAGAAATGAAGTTGGTAACAAAACCATGGCAGAACCAAATCAAATAAGCGGTTTGGCAGGTTGGCATTCTTGAGGGAAGAACTTTCAGCATTCTTGGGCAACTACGGTAGAATCTCGTCCCTTTTTGTTCCCCTTTTGGATTGGTCTTTCAACGGAGAATCTCCTTCATCCAGGCCCTACCACTTCCCCCACTAGGAATTTATCCCCACGCCTCGTTGCTGCAAGCAAGCATCGGAATATAATCTGGCAGTAGTAGTAGTGGTACGAAAATAACCACACACCTACGCGGCTACACCAAACATCAGAAAAAATGCAGGCGATGCATGATTTATTAGGAAATGTCAGGCAAAGACTAAGAATCGAACAATGCGATAAGTCTCTCGAGAAAGAAATCGCAACGTGATACACATAACCTTATTCGTCAGAGAATGTGACACGCACTTGCTGACTAGCGTGTAGATGCCTGAAAATTCAGCTCGCCCTCCACCAAGAAGAAGATAAGATTCAGATCAGCGCCCCCTCGCAGCCTGCAGATGAAGACGAATAAAGCCACGAGATGCTGCGCAGATCGGATCGACTATAAAAGGAGGCGCAGCCGCGAGAGGCCCTTACACCATCCATCACCTTAGCTAGCATAGCATCCCGTCTCTGCGTCCTGAGCTTGCGAACGCGGCCGGACAT

Promotor *TraesCS5D02G238700* chr5D:346 688 696..346 689 494

chr5D:346 689 406..346 690 887

TTTGTCTGTGCCTGTTTCCTCTGCTCTTGTTGCCTTGGTGCTTGCTTCTTGATCGTCTCTGAGATGAGAATTGTCGCTGCGTGCTGGATGCTATTTGCCGAGCCCGAAGGCTCGGGATATATAGTCCCGGGTTGCCTCGCGAGAAGGAGGGAAAGAAGTTGGAAGGGGGGACGGACGACGGCCGGGACTTGGGAAGAAAGCCGGGAGCGAGGAAGGTTCCCGGAAGCAGGGAGGCCTGGTGGAAACGCCGGACCGACGGAGCGGTCGCCGGTTCGTGCCTTGTGAGAGCGATGGAATTGTCGCGCGCGCGCGCGAGCGGGTTTCGAGGGAGCTTCGCGGCGTGGAAACATGGGCGGGTCGACGTCGGTCGTCTCGTCACGGCGCACCGGCGACCCGACCGTATTCT**[C/T]**GGTTGGGGTTGGCGTTGGAATTCCACGCAGCCTCGCCTGCTACTCTTTCCGTTGTCGGGTTCCTCCTGGTTGATTTTCCTTCTGCGACTGAATTTTGATTTGATTTCGATAGATATATGTACGGTGGTGGACTGGCCGCACGGACCTGGTGGTATACGTGTTCAAGAAAAATTAGACATCGTGTGTGTTTGTGTGTGACCTGGTGGTATACGGCCACTGGCGTGAAGGAAGGTTGGTGAGTTGGCTAGCGAGCTCTTGACCCGATTTCGTGGTGGACTCGACCAAACCAAACCAAACCAAGCAGTGGTGTAGGTCTTCGCGCCAGCGGACGTGAGAGAATGCTTGATAATGCTTTTTTCCCAGCAAATTTCCAATCTATTCATCTTCAATCATGATAATACCATGAATATAAAAAATAATAAAAAATACATTCAGATTCATAAACCACCTAGTGACGACTATAAGCACTGAAGCATGCTGAAGGCGC**[G/A]**CCGCCGTCATCGCCCCTCTGTTGCCGAAGTTGGGCACAACTTGTTAGTGAGAAAGTCGTCGTGCTAAATCTCCATAAGACGAGCGCACCAGAGCAACAACCGCCGCTGATGAAAAAAAAACGTAAATCGAAAGGATTCAACTTGAAGACACACGATCGTAGACGAACAACGACGAGATCCGAGCAAATCCATCAAAGATAGATTCGCCGGAGACACACCTCCACATGTCCATCAATGATGCTAGGCACACCGCCAGAACGGGCGCTAAACAGGAAAACCTTTATTTCATCTTCAGGGACCGCCGCCGTCTCGCTTTAATGAGCAGGACATAAACCATAACAAAACCGAAAGGGACGACTAAAATCGGAGCTCTCCCGCTAGCCCTTGCGAGGATCCACCACGCTCTCATGACCCTAGGCCCACTGAAGATGAGGCGGACCTACGACAACACCGATGAGAGACATGAACCCTAGCCTTTTTTAAGAAGGAGGCGGCTATACATGATCGTGCTTCCACCACCGTCCACTATGACTGA**[/G]**GGTGTTTACACGCGACTAATGTGCTAATTATGTGTGCGAGCGGGTGCATATA
